# Standardized numbering and alignment of the KPC family of β-lactamases

**DOI:** 10.1128/aac.01868-25

**Published:** 2026-05-29

**Authors:** Florencia Brunetti, Steven H. Marshall, Christopher R. Bethel, Andrea M. Hujer, Kristine M. Hujer, Shozeb Haider, Karen Bush, Patricia A. Bradford, Barry Kreiswirth, James Spencer, Yoshikazu Ishii, Jean-Denis Docquier, Gian Maria Rossolini, Laurent Poirel, Mariagrazia Perilli, Gianfranco Amicosante, Moreno Galleni, Alejandro J. Vila, Gabriel Gutkind, Timothy Palzkill, Alessandra Carattoli, Catherine L. Tooke, Philip Hinchliffe, Christopher J. Schofield, Bogdan I. Iorga, Thierry Naas, Yu Chen, Cesar Arias, Andrea Endimiani, Krisztina Papp-Wallace, Ryan K. Shields, Pranita D. Tamma, Robert A. Bonomo, Pablo Power

**Affiliations:** 1 Instituto de Investigaciones en Bacteriología y Virología Molecular (IBaViM), Facultad de Farmacia y Bioquímica, Universidad de Buenos Aires 28196, Buenos Aires, Argentina; 2 Research Service, Louis Stokes Cleveland Department of Veterans Affairs Medical Center 20083, Cleveland, Ohio, USA; 3 Department of Medicine, School of Medicine, Case Western Reserve University 2546, Cleveland, Ohio, USA; 4 UCL School of Pharmacy, University College London 4919, London, United Kingdom; 5 Biology Department, Indiana University 1772, Bloomington, Indiana, USA; 6 Antimicrobial Development Specialists, Nyack, New Yorl, USA; 7 Center for Discovery and Innovation, Hackensack Meridian Health Inc 3139, Nutley, New Jersey, USA; 8 School of Cellular and Molecular Medicine, University of Bristol 1980, Bristol, United Kingdom; 9 The Center for Planetary Health and Innovation Science (PHIS), The IDEC Institute, Hiroshima University 12801, Hiroshima, Japan; 10 Dipartimento di Biotecnologie Mediche, Università di Siena 574285 , Siena, Italy; 11 Department of Experimental and Clinical Medicine, University of Florence, and Microbiology and Virology Unit, Careggi University Hospital 18561 , Florence, Italy; 12 Medical and Molecular Microbiology, Faculty of Science and Medicine, University of Fribourg 27211, Fribourg, Switzerland; 13 Department of Biotechnological and Applied Clinical Sciences, University of L’Aquila, L’Aquila, Italy; 14 Laboratory of Enzymology and Protein Folding, Centre for Protein Engineering, InBioS, University of Liege, Liege, Belgium; 15 Instituto de Biología Molecular y Celular de Rosario (CONICET IBR -UNR) 63031 , Rosario, Argentina; 16 Consejo Nacional de Investigaciones Científicas y Técnicas (CONICET) 62873, Buenos Aires, Argentina; 17 Verna and Marrs McLean Department of Biochemistry and Molecular Pharmacology, Baylor College of Medicine 3989, Houston, Texas, USA; 18 Department of Molecular Medicine, Sapienza University of Rome 9311, Rome, Italy; 19 Department of Life Sciences, University of Bath 1555, Bath, United Kingdom; 20 Chemistry Research Laboratory, Department of Chemistry and the Ineos Oxford Institute for Antimicrobial Research, University of Oxford 6396, Oxford, United Kingdom; 21 Université Paris-Saclay, CNRS, Institut de Chimie des Substances Naturelles, Gif-sur-Yvette, France; 22 Team ReSIST, Université Paris-Saclay 27048, Gif-sur-Yvette, France; 23 Department of Molecular Medicine, University of South Florida 7831, Tampa, Florida, USA; 24 Department of Medicine, Weill Cornell Medical College 12295 , New York, New York, USA; 25 Institute for Infectious Diseases (IFIK), University of Bern 27210, Bern, Switzerland; 26 Department of Biochemistry, School of Medicine, Case Western Reserve University 2546, Cleveland, Ohio, USA; 27 Department of Medicine, University of Pittsburgh 6614, Pittsburgh, Pennsylvania, USA; 28 Department of Pediatrics, University of Pennsylvania Perelman School of Medicine 14640 , Philadelphia, Pennsylvania, USA; 29 Departments of Molecular Biology and Microbiology, Pharmacology, and Proteomics and Bioinformatics, School of Medicine, Case Western Reserve University 2546, Cleveland, Ohio, USA; 30 CWRU-Cleveland VAMC Center for Antimicrobial Resistance and Epidemiology (Case VA CARES) USA, Cleveland, Ohio, USA; Entasis, Big Bay, Michigan, USA

**Keywords:** KPC INDEL variants, KPC variants, KPC carbapenemase, beta-lactamase nomenclature

## Abstract

The KPC family of serine β-lactamases comprises more than 260 members. Some variants are associated with ceftazidime-avibactam resistance in clinical isolates, often linked to substitutions and/or insertions/deletions (i.e., INDELs) in three distinct loops of the KPC sequence: (i) the 164–179 loop (i.e., the Ω loop); (ii) the 237–243 loop; and (iii) the 267–275 loop. Inconsistencies in residue numbering across published reports, however, complicate the accurate annotation of KPC variants. We retrieved 267 KPC variant sequences from the Beta-Lactamase Database (BLDB) in September 2025 and analyzed sequence differences between variants using combined nucleotide and structure-guided alignment algorithms, supported by AlphaFold3 modeling in ambiguous cases. Variants were classified into four groups to comprehensively review sequence changes across the KPC family: substitutions only (*n* = 126), deletions only (*n* = 17), insertions only (*n* = 66), and variants with two or more types of amino acid changes (*n* = 57). Comparisons with previous reports indicate that many annotation errors stem from overlooking the absent residues 58 and 253 in the KPC consensus sequence, and that most inconsistencies in annotation and residue assignment occur in INDEL variants. To address these issues, we propose a standardized annotation scheme for substitutions, deletions, and insertions for the KPC family, based on the Ambler numbering system, and supported by structural information. This systematic scheme will help to standardize the description of newly emerging KPC variants and prevent discrepancies in future reports in the context of antimicrobial resistance.

## INTRODUCTION

The first report of *Klebsiella pneumoniae* carbapenemase (KPC) came from a *K. pneumoniae* isolated in the United States in the late 1990s. It was designated as KPC-1 (1, 2). Shortly thereafter, a variant having a single amino acid substitution was described in *Klebsiella* spp. and *Salmonella enterica* and was named KPC-2 (3–5). However, a few years later, an erratum letter described an unfortunate sequencing error in the “original” *bla*_KPC-1_ sequence, which made the genes encoding for KPC-1/2 identical (6). For convenience, as other novel variants had been described as KPC-2-derived variants (KPC-3 and KPC-4), the KPC-2 designation replaced KPC-1 permanently (7, 8). KPC-producing isolates are now found worldwide, with *bla*_KPC_ genes identified in Enterobacterales, *Pseudomonas*, and *Acinetobacter*.

KPC-2 is the most prevalent class A serine carbapenemase, with its dissemination largely linked to *K. pneumoniae* clonal complex 258 (CC258) (9). CC258 includes the successful sequence type 258 (ST258) lineage—the primary driver of outbreaks in the Americas, Europe, and Israel—and related STs, such as ST11 (dominant in China), ST340, and ST512, among others (9, 10). More recently, however, ST307 (which belongs to CC307) and ST147 have emerged as new high-risk clones furthering the dissemination of *bla*_KPC_ genes (11–13).

**Fig 1:**
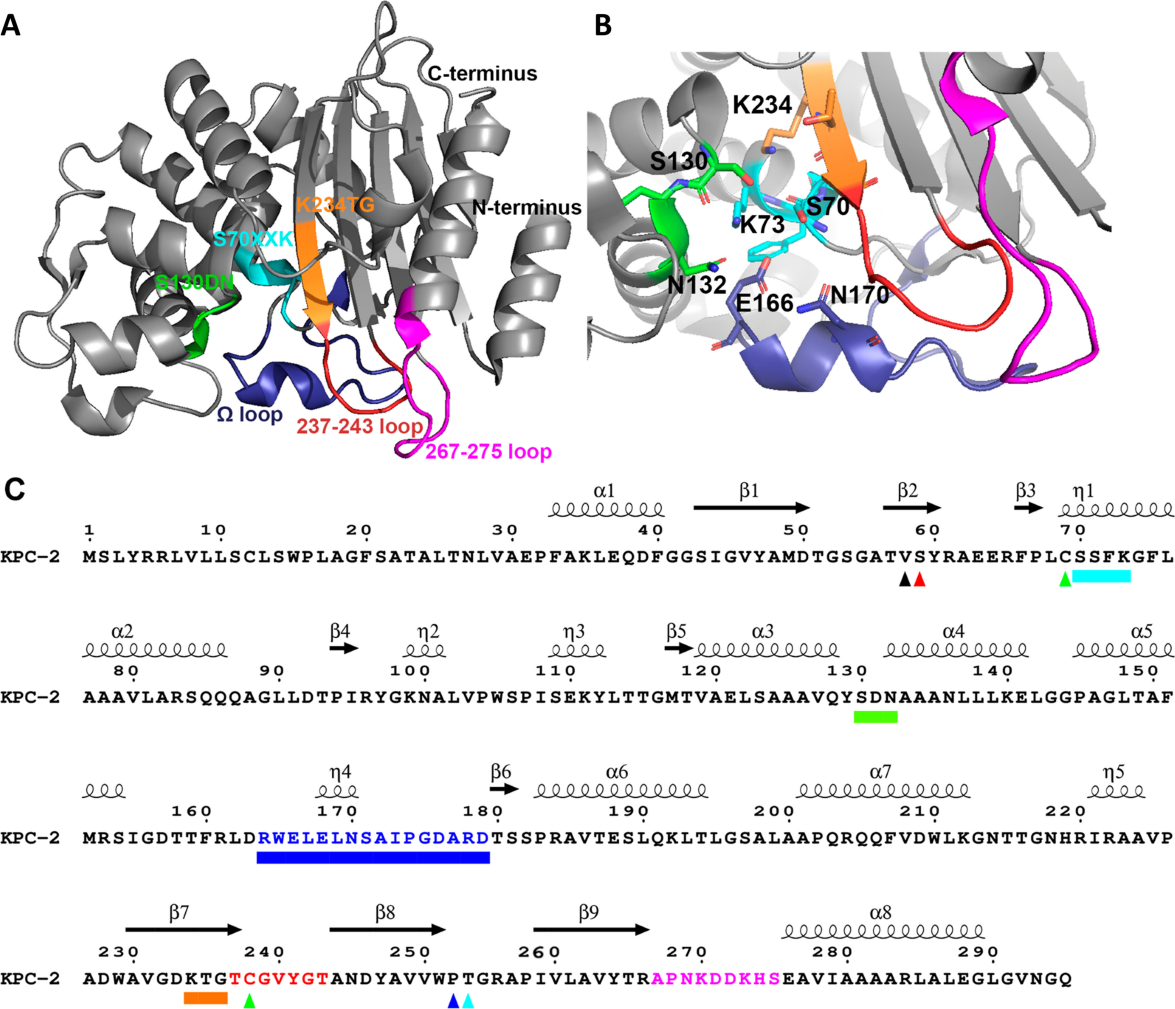
(**A**) KPC-2 structure (PDB: 2OV5) showing conserved amino acid motifs (cyan: S^70^XXK; green: S^130^DN; blue: Ω-loop, amino acids 164–179; orange: K^234^TG) and loops associated with CZA resistance (blue: Ω-loop; red: 237–243 loop; pink: 267–275 loop). (**B**) Relevant residues in the KPC-2 active site involved in β-lactamase activity. (**C**) Amino acid sequence of KPC-2 following Ambler numbering. The sequence highlights conserved amino acid motifs and loops associated with CZA resistance, using the color scheme previously defined. Secondary structure elements (derived from crystallographic structure PDB 2OV5) are shown above the sequence. The required numbering adjustment to match the Ambler scheme are marked with triangles (V57: black triangle; S59: red triangle; P252: blue triangle; T254: cyan triangle). The disulfide bridge between C69 and C238 is highlighted with green triangles.

The KPC-2 β-lactamase belongs to Ambler class A and the Bush-Jacoby-Medeiros functional group 2f (14). The active site of KPC contains the four conserved amino acid motifs characteristic of class A β-lactamases (S^70^XXK; S^130^DN; amino acids 164–179 named as Ω-loop; and K^234^TG; Fig. 1A through C), but also has unique structural features, including an expanded active site that enables hydrolysis of a broad spectrum of β-lactams, including penicillins, most cephalosporins, monobactams, and carbapenems (3, 15). Notably, KPC-2 is not effectively inhibited by the widely used β-lactam-based β-lactamase inhibitors (BLIs), because it can hydrolyze clavulanic acid, sulbactam, and tazobactam (16). This broad hydrolytic profile results in resistance or reduced susceptibility to nearly all β-lactams in KPC-2-producing isolates. Furthermore, these isolates frequently harbor additional resistance genes, leading to multidrug-resistant phenotypes (3). However, infections caused by KPC-2-producing isolates can be successfully treated with combinations containing non-β-lactam β-lactamase inhibitors, such as the diazabicyclooctane inhibitors (DBOs), including ceftazidime-avibactam (CZA) and imipenem-relebactam (IMR), and boronic acid inhibitor combinations, such as meropenem-vaborbactam (MEV) (17–20). Notably, these BLIs were primarily designed to target KPC hydrolytic activity (17–19).

The KPC family currently includes 267 identified variants (Beta-Lactamase Database [BLDB], September 2025) (21). The first documented KPC member was KPC-2 (formerly misnamed KPC-1) (2), and the first reported variant was KPC-3, which contains the H274Y substitution that confers a 30-fold increase in efficiency of ceftazidime hydrolysis compared to KPC-2 (22). Notably, multiple amino acid changes in the KPC β-lactamase have been associated with CZA resistance, suggesting that selective pressure from this agent drives KPC diversification (23, 24).

Structural analyses have identified that the amino acid changes conferring a CZA resistant phenotype are mostly located in three “hotspots” in the KPC β-lactamase sequence: (i) the Ω loop region (amino acids 164–179); (ii) the 237–243 loop; and (iii) the 267–275 loop (Fig. 1A through C) (23). Prior to this study, different types of amino acid changes have been identified in clinical isolates for each loop: while substitutions (including the clinically relevant D179Y substitution), deletions, and insertions are observed within the Ω-loop (23, 25, 26), the 237–243 loop had only substitutions and deletions, and the 267–275 loop exclusively exhibited substitutions and insertions (23). KPC deletions and insertions involve one to several amino acids, and insertions can include duplications of up to 28 residues (23). Furthermore, some KPC variants exhibit simultaneous modifications in multiple loops (23).

The extensive diversification of the KPC family has led to numerous reports documenting the detection of novel variants and the analysis of their biochemical and structural properties (23, 27–38). Many of these analyses, however, describe amino acid substitutions and/or insertion/deletion (INDEL) modifications according to a sequential residue numbering for new variants, a method which can lead to misunderstanding. Firstly, it may result in errors in identifying the exact location of amino acid changes. Secondly, it can misalign conserved residues that are essential for hydrolytic activity. To address these issues, we propose a standardized KPC variant numbering scheme based on the use of structure-based alignment tools, supported by *in silico* or experimental (i.e.*,* X-ray diffraction) structures. The scheme establishes consensus in the description of KPC variants and will facilitate the precise annotation of substitutions, deletions, or insertions in future variant submissions.

## RESULTS AND DISCUSSION

### KPC-2 numbering scheme

According to the standardized numbering scheme proposed by Ambler (39), functionally relevant residues (critical for hydrolytic activity) in class A β-lactamases are consistently numbered across protein sequences within this group. Important conserved residues include S70 (the reactive serine residue responsible for the nucleophilic attack on the β-lactam ring’s carbonyl moiety) and K73 (conserved motif “SXXK”); S130-D131-N132 (conserved motif “SDN”); K234-T/S235-G236 (the conserved “KT/SG” triad); and E166 in the Ω loop, essential for hydrolysis of the β-lactam-derived acyl-enzyme complex and subsequent regeneration of activity (Fig. 1A through C). Other residues, not strictly conserved in all class A β-lactamases, but relevant for activity, include Y/W105, H/N170 in the Ω loop, R220 (the equivalent of R244 in, e.g., TEM/SHV enzymes), and T/S237. We therefore aligned these residues precisely as benchmarks for adjusting residue numbering (Fig. 1C).

When a sequential numbering system is applied to the KPC-2 sequence, conserved residues important in catalysis are misassigned. For example, the nucleophilic serine (Ambler position S70) appears as residue 69 in KPC-2 when counting from the N-terminal methionine. This inconsistency has already been addressed in the crystallographic structure of KPC-2 (2OV5) by creating a “jump” in the sequence numbering between V57 and S59, for which “residue 58” is lacking (Fig. 1C) (15). In other class A β-lactamases, such as TEM-1 and SHV-1, a residue at position 58 is present (E58 and T58, respectively). Some standard alignment algorithms fail to account for the missing residue at position 58. We propose that inserting a virtual residue (e.g., a dash “–”) between V57 and S59 in the KPC-2 sequence prior to alignment, or manually adjusting the numbering post-alignment, will resolve this issue and serve as a standardized approach for future analyses. In this study, we implemented the second approach using the ESPript 3.2 server, as described in the Materials and Methods section.

Another issue in the KPC numbering scheme is a “jump” in the residue numbering from 252 to 254, which makes residue 253 “disappear” from the numbered sequence of KPC-2 (2OV5) and KPC-3 (6QWD). After carefully checking different structures of other class A β-lactamases in the Protein Data Bank (PDB), such as TEM-1 (1BTL), SHV-1 (1SHV), CTX-M-96 (3ZNY), NMC-A (1BUE), and SME-1 (9JNC), we observed that they all uniformly include this same jump. Interestingly, the crystal structure of the PC1 β-lactamase from *Staphylococcus aureus* (3BLM) includes a residue 253 in the sequence, and the first structure of TEM-1 was solved by molecular replacement from the coordinates of PC1 (40, 41). Therefore, it is very likely that this discontinuity at position 253 originated from that early comparison to the PC1 sequence. Although this shift in the numbering has generated discrepancies throughout the literature, we propose to keep the relevant histidine residue—substituted by tyrosine in the KPC-3 β-lactamase (and its derivatives)—as H274 (Fig. 1C). This nomenclature is also the most used among sequences and structure coordinates in databases.

### KPC variants with substitutions only

**Fig 2:**
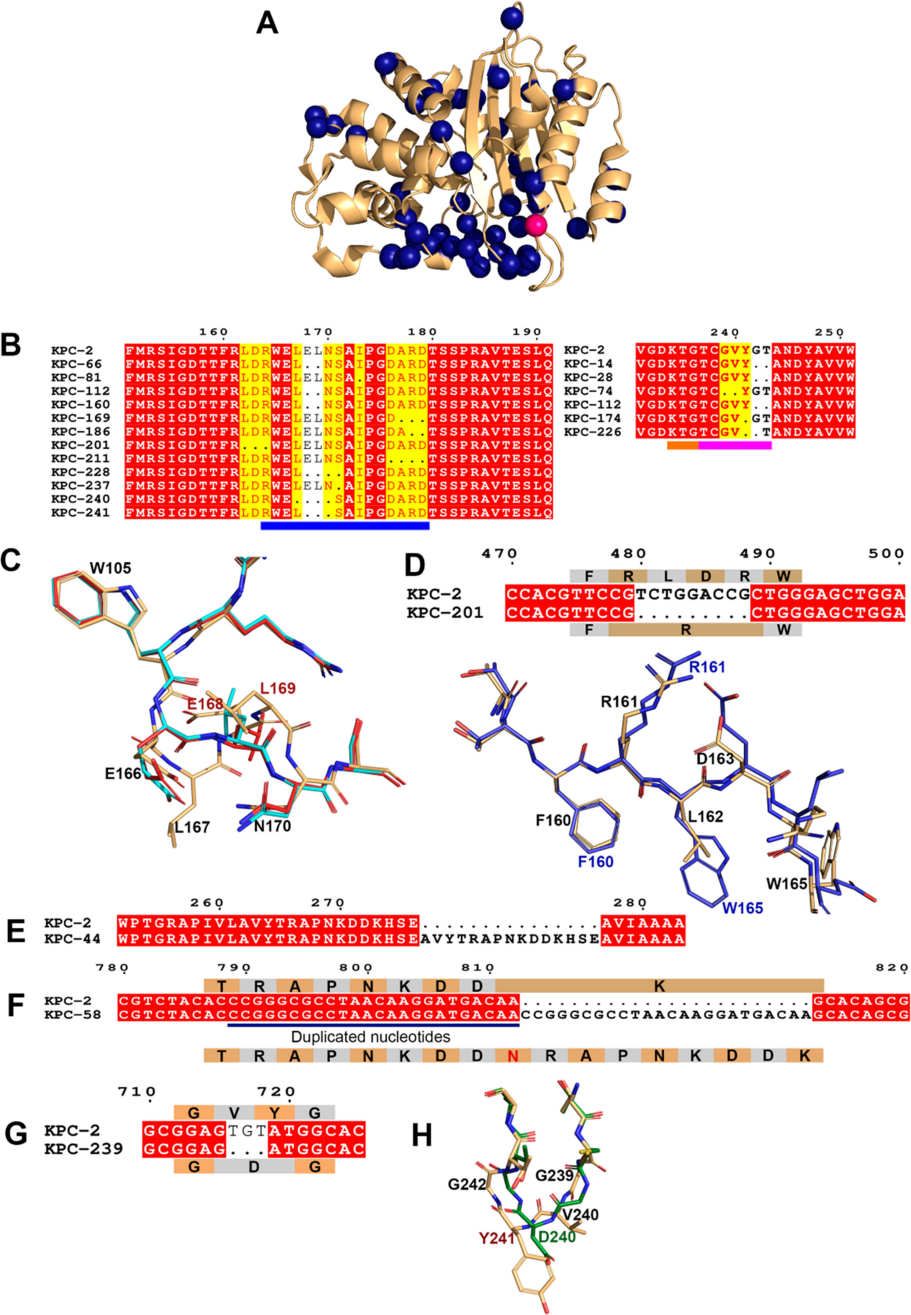
(**A**) Location of amino acid substitutions (blue spheres) observed in 126 KPC variants harboring only substitutions, mapped onto a KPC-2 crystal structure (PDB: 2OV5, backbone cartoon colored tan). Substitution at residue H274 (H274Y) gives rise to KPC-3 and derived variants (magenta sphere). (**B**) Multiple sequence alignment of the 17 KPC variants containing only deletions. The Ω loop is highlighted in blue (left), the K234-T235-G236 motif in orange, and the 237–243 loop in fuchsia (right). (**C**) Superposition of the KPC-2 structure (PDB: 2OV5, tan) with *in silico* models of KPC-66 (red) and KPC-160 (cyan) obtained using AlphaFold3. Residue numbering follows the KPC-2 sequence; the residues proposed to be deleted in KPC-66 and KPC-160 (E168 and L169) are highlighted in red. (**D**) (Top) Nucleotide alignment of KPC-2 and KPC-201, showing the out-of-frame deletion (positions 480–488) in KPC-201. Boxes indicate the corresponding amino acid residues translated from each codon for both KPC variants. (Bottom) Structural superposition of KPC-2 (PDB: 2OV5, carbon atoms tan) with the KPC-201 *in silico* model (carbon atoms blue) predicted by AlphaFold3. Residue numbering in black corresponds to KPC-2, while blue numbering indicates KPC-201 positions. (**E**) Structural alignment of KPC-2 and KPC-44 amino acid sequences, highlighting the 15-residue insertion (AVYTRAPNKDDKHSE) between positions E276 and A277 in KPC-44. (**F**) Nucleotide alignment of KPC-2 and KPC-58 showing the out-of-frame insertion after position 812 (adenine) of the duplicated 788–812 nucleotides in KPC-58. This insertion generates the D272_ins(NRAPNKDD)_K273 modification in the 267–275 loop, where the frameshift results in translation beginning with residue N272a (absent in KPC-2). Boxes indicate the corresponding translated amino acid residues for both variants. (**G**) Nucleotide alignment of KPC-2 and KPC-239 showing the out-of-frame deletions that simultaneously generate a contiguous V240D substitution and Y241 deletion. (**H**) Structural superposition of KPC-2 (PDB: 2OV5, tan) with the KPC-239 *in silico* model (green) predicted by AlphaFold3. Residue numbering in black corresponds to KPC-2, while green numbering indicates KPC-239 residues. The Y241 residue proposed for deletion is shown in red.

Among the KPC β-lactamase family sequences, 126 variants contained substitutions exclusively, establishing this as the most prevalent amino acid modification type (Fig. 2A). The predominant substitution was H274Y within the 267–275 loop, a substitution that defines KPC-3 and its derivatives (at present, 83 variants possess this substitution in the KPC family).

Beyond the H274Y change, substitutions occurred most frequently in the Ω loop. D179 was the most frequently substituted Ω-loop residue, generating clinically important variants including KPC-31 and KPC-33 (23, 42), which were the first substitutions reported following treatment with CZA (43). The 237–243 loop exhibited substitutions at four positions (V240, Y241, T243, and A244). Within, and adjacent to, the 267–275 loop, substitutions were observed at Y264, T265, P268, and E276, in addition to the frequently observed H274Y substitution. Notably, substitutions also occurred at various positions outside these three loops (*n* = 44). Furthermore, 31 variants exhibited more than one substitution (in addition to H274Y), with 21 of these variants displaying this type of alteration at multiple spots.

When residue numbering follows the Ambler scheme and the absent residues 58 and 253 in KPC sequences are considered, identifying the exact location of amino acid substitutions does not present significant challenges. Nevertheless, our review revealed several reported KPC variants containing misnumbered amino acid changes as a consequence of the “missing” residues 58 and 253. Representative examples of these discrepancies are listed in Table 1. These observations highlight the critical importance of applying residues 58 and 253 in sequence alignments to ensure accurate identification and reporting of substitution positions in KPC variants according to the Ambler scheme. The amino acid alignments for all 126 substitution variants are included in the Supplemental material.

**TABLE 1:** Annotation proposal for representative KPC variants containing amino acid substitutions exclusively

KPC variant	Accession number	Reported amino acid substitutions	Proposed annotation^*a*^
KPC-3	WP_004152396.1	H272Y	**H274Y**
KPC-4	WP_015062847.1	P103R, V239G	**P104R, V240G**
KPC-62	WP_204376229.1	L168Q	**L169Q, H274Y**
KPC-70	WP_210205477.1	D179Y, T268A	**D179Y, T265A, H274Y**
KPC-145	WP_268871853.1	T263A	**D179Y, T265A**

^*a*^ Bold terms indicate the proposed annotation.

### KPC variants with deletions only

Of the 267 sequences analyzed, only 17 KPC variants contained deletions, and all were located within either the Ω loop or the 237–243 loop (Fig. 2B). Our review revealed inconsistent nomenclature for describing deleted amino acids in KPC variants. To standardize reporting, we propose the following naming convention for further description of KPC deletions: “First flanking amino acid_∆deleted residues_Next flanking amino acid.” Thus, for example, in KPC-14 where a glycine (G242) and a threonine (T243) are deleted, this mutation would be designated as “Y241_∆GT_A244” according to our proposed scheme.

Overall, significant challenges were not encountered using structure-based alignment (based on the Ambler numbering scheme) for determining the deletion positions in the 237–243 loop, as previously mentioned.

However, there are issues with Ω loop deletions, particularly in the “E166-LEL” region, which is coded by the tandem nucleotides “GAGCTGGAGCTG.” When deletions in this region involve two residues containing the repeated “EL” sequence, as observed in the KPC-66 and KPC-160 variants, sequence alignments with the MAFFT algorithm suggested deletion of E166 and L167. On the other hand, structural alignment results obtained with the T-Coffee Server, using the KPC-66 and KPC-160 sequences aligned with KPC-2 suggest that the deletion involves L167 and E168. This result remained consistent when including other variants with similar two-residue deletions (KPC-73, KPC-112, KPC-124, KPC-135, KPC-197, KPC-238). However, in some iterations where we included KPC sequences with different types of mutations, we found that the suggested deletions in this area affect E168 and L169, as reported (32).

We analyzed all variants with deletions in the “E166-LEL” region (Fig. 2B) and observed that the E166L167 pair of residues is best represented as a repeating unit that appears twice in KPC-2, or once when a deletion of an EL pair occurs, as in KPC-66 and KPC-160. Therefore, residue L167 is consistently present in these variants shown in Fig. 2B, except in KPC-240, which retains only the first E166. From a mechanistic perspective, given that these variants may result from a 6-nucleotide deletion (i.e., two codons), E166 and L167 should be regarded as the conserved residues, although AlphaFold3 modeling may lead to ambiguous predictions of the spatial position of the tandem EL residues (Fig. 2C). Therefore, for variants carrying double deletions in the E166-LEL region (such as KPC-66, KPC-73, KPC-112, KPC-124, KPC-135, KPC-160), we propose that these mutations should be designated as “L167_∆EL_N170” in agreement with our naming convention.

Another issue we identified is that some deletions do not always occur in-frame at the nucleotide level. As an example with KPC-201, the MAFFT algorithm suggests that this variant has the deletion of “R161-L162-D163”; however, structural alignment indicates deletion of “L162-D163-R164.” Nucleotide analysis reveals that the deletion extends from the third base of the R161 codon to the second base of the R164 codon, with the remaining bases forming a new codon for arginine (Fig. 2D). The *in silico* modeling obtained with AlphaFold3, structural alignment, and the nucleotide analysis suggests that the “R161_∆LDR_W165” deletion occurs, since the remaining R aligns with residues R161 in the KPC-2 structure (Fig. 2D). Although the confidence scores for the models obtained with AlphaFold3, measured by predicted local distance difference test (pLDDT), were insufficient to reliably assess the positions of the side chains around the deletion, we propose the consensus annotation “R161_∆LDR_W165*”* for the KPC-201 variant.

Our results demonstrate that multi-sequence alignment programs for amino acid numbering should generally be avoided, as they may produce inconsistent results depending on the specific sequences included and the specific programs used. Based on our findings, we recommend performing individual, structurally informed alignments against KPC-2 for individual variants in order to prevent potential biases introduced by including additional sequences in multi-sequence alignments. Furthermore, our findings indicate that standard amino acid alignment algorithms lacking structural considerations, such as MAFFT, may inaccurately identify residues (as proposed herein) involved in deletions, compared to algorithms that include structural information, like the T-Coffee Expresso server employed in this study. We emphasize the importance of complementary nucleotide alignment to assess out-of-frame deletions and, in the absence of experimental data, recommend the use of *in silico* models for structural insights. Table 2 presents our proposed system for deletion numbering for KPC variants.

**TABLE 2:** Annotation proposal for representative KPC variants containing amino acid deletions exclusively

Location	KPC variant	Accession number	Proposed annotation
Ω loop	KPC-66	WP_188331871.1	**L167_∆EL_N170, H274Y**
	KPC-81	WP_204376234.1	**A172_∆I_P174**
	KPC-160	WP_279240777.1	**L167_∆EL_N170**
	KPC-169	WP_338424102.1	**G175_∆DAR_D179**
	KPC-186	WP_311033309.1	**A177_∆RD_T180**
	KPC-201	WP_305449896.1	**R161_∆LDR_W165, H274Y**
	KPC-211	WP_336433394.1	**G175_∆DARD_T180**
	KPC-228	WP_348866574.1	**L167_∆ELNS_A172**
	KPC-237	WP_394294903.1	**N170_∆S_A172**
	KPC-240	WP_394294906.1	**E166_∆LELN_S171, H274Y**
	KPC-241	WP_394294907.1	**L167_∆ELN_S171**
237–243 loop	KPC-14	WP_063860636.1	**Y241_∆GT_A244**
	KPC-28	WP_072081992.1	**Y241_∆GT_A244, H274Y**
	KPC-74	WP_188331874.1	**C238_∆GV_Y241**
	KPC-174	WP_338424106.1	**V240_∆Y_G242**
	KPC-226	WP_367187954.1	**V240_∆YG_T243**
Ω loop, 237–243 loop	KPC-112	WP_240067722.1	**L167_∆EL_N170, Y241_∆GT_A244**

### KPC variants with insertions only

We identified 66 KPC variants containing only insertions. These insertions ranged from single amino acids to over 28 residues (e.g., KPC-68 and KPC-107, respectively). Although some insertions represented exact tandem duplications of existing residues in the KPC sequence, others did not show exact duplication patterns. The nucleotide alignment of these latter cases revealed that they resulted from out-of-frame insertions (e.g., KPC-58 and KPC-93), highlighting the importance of conducting both amino acid and nucleotide-level alignments to properly characterize insertion positions.

As for our findings with deletions, we observed inconsistencies in how insertions were annotated across different reports. Again, as the genetic mechanism involved in the tandem amino acid duplications is not known, we propose a convention annotation scheme where inserted residues are listed immediately after the last duplicated amino acid. For example, KPC-44 contains a 15-residue tandem insertion (AVYTRAPNKDDKHSE), where the last duplicated residue is E276 (44) (Fig. 2E). According to our proposed scheme, this would be annotated as “E276_ins(AVYTRAPNKDDKHSE)_A277.” For numbering the inserted residues, we suggest using the last duplicated residue’s number followed by alphabetical designations. In the case of KPC-44, the inserted residues will be numbered as follows: A276a, V276b, Y276c, T276d, R276e, A276f, P276g, N276h, K276i, D276j, D276k, K276l, H276m, S276n, and E276o. When insertions exceed all usable alphabet letters, as in KPC-107, we recommend continuing the numbering by using “aa,” “ab,” “ac,” and so on. For practical reporting of large insertions, we propose abbreviated annotations specifying the number of residues inserted, for example, “E276_ins (15)_A277” for KPC-44.

Until now, insertions have only been reported within either the Ω-loop or the 267–275 loop (23). Our analysis identified two variants with insertions located adjacent to the 237–243 loop: KPC-263 (G239_ins(G)_V240) and KPC-252 (A248_ins(AANDYA)_V249). The “G239_ins(G)_V240” insertion was also found in KPC-141, which carries the D179Y substitution. Additionally, when examining all 66 KPC variants included in this subgroup, we observed that insertions result from exact duplication of nucleotides located immediately adjacent to the insertion site. These duplicated bases can be inserted either in-frame, generating tandem amino acid duplications, or out-of-frame, producing insertions beginning with residues absent in the original KPC-2 sequence. To accurately identify the duplicated nucleotides, we implemented a workflow involving: (i) ClustalW alignment of the variant nucleotide sequence with KPC-2; (ii) translation of the pre-aligned nucleotide sequences; and (iii) structural validation through comparison with alignments obtained with T-Coffee Expresso server.

In general, the out-of-frame insertions were correctly detected when using combined nucleotide/amino acid alignments. For example, in KPC-58, we observed a duplication of nucleotides 788–812 that were inserted out-of-frame after the adenine at position 812, resulting in an insertion initiating with N272a, a residue absent in KPC-2 (Fig. 2F).

We encountered difficulties with tandem duplication insertions, as in KPC-44, because both standard and structural alignment algorithms frequently misassigned the insertion boundaries, producing varying results depending on the input sequences and launch parameters. Therefore, we recommend studying each KPC sequence separately rather than including them in a multi-alignment and manually observing and curating the parameters to detect potential errors introduced by the initial algorithm. This manual verification can be challenging since tandem insertions containing many amino acids may further complicate the determination of exactly where the duplicated residues begin and end. For instance, the structural alignment suggested that the residues duplicated in KPC-44 were YTRAPNKDDKHSEAV, but careful examination of the sequence revealed this was inaccurate. Indeed, the tandem duplication most likely begins at A262, since L261 (the residue immediately preceding the duplicated segment) does not appear in the sequence of duplicated residues (Fig. 2E).

We also identified ambiguities in the “E166LEL” region of the Ω-loop, where certain variants (e.g., KPC-25, KPC-53, and KPC-152) exhibit duplications of two residues. Structural alignments could not determine which residues were duplicated in tandem. For these cases, we propose adopting the convention of E166L167 duplication, which considers the E166L167 pair of residues as the conserved unit that appears three times in these variants. As per our proposed annotation scheme, this would be designated as “L167_ins(EL)_E168,” with the final resulting sequence being “E166-L167-E167a-L167b-E168-L169-N170.”

Our analysis identified that, for some variants, the residues involved in insertions are inaccurately reported due to improper identification of the duplicated nucleotides and the positions of these insertions. Selected examples of annotations for insertions observed in this variant group are presented in Table 3.

**TABLE 3:** Annotation proposal for representative KPC variants containing amino acid insertions exclusively

Location	KPC variant	Accession number	Proposed annotation^*a*^	Abbreviated annotation
Ω loop	KPC-25	WP_065419571.1	**L167_ins(EL)_E168**	L169_ins (2)_N170
	KPC-53	WP_156649232.1	**L167_ins(EL)_E168, H237Y**	L169_ins (2)_N170, H237Y
	KPC-71	WP_194293134.1	**S181_ins(S)_S182**	–^*b*^
Near the Ω loop	KPC-114	WP_242934069.1	**S182_ins(SS)_P183**	S182_ins (2)_P183
267–275 loop	KPC-29	WP_096807439.1	**D272_ins(KDD)_K273, H274Y**	D272_ins (3)_K273, H274Y
	KPC-41	WP_148044419.1	**K270_ins(PNK)_D271, H274Y**	K270_ins (3)_D271, H274Y
	KPC-58	WP_179284320.1	**D272_ins(NRAPNKDD)_K273**	D272_ins (8)_K273
	KPC-67	WP_210204487.1	**D272_ins(KDDKDD)_K273, H274Y**	D272_ins (6)_K273, H274Y
	KPC-79	WP_197749404.1	**N269_ins(VYTRAPN)_K270**	N269_ins (7)_K270
	KPC-80	WP_204376233.1	**K270_ins(PNK)_D271**	K270_ins (3)_D271
	KPC-82	WP_202781289.1	**S275_ins(DS)_E276**	S275_ins (2)_E276
	KPC-109	WP_274293083.1	**H274Y_ins(NKDDKY)_S275**	H274Y_ins (6)_S275
	KPC-113	WP_242934068.1	**R266_ins(G)_A267**	–
Near the 267–275 loop	KPC-34	WP_109545044.1	**A277_ins(KDDKHSEA)_V278**	A277_ins (8)_V278
	KPC-50	WP_171476788.1	**H274Y, V278_ins(EAV)_I279**	H274Y, V278_ins (3)_I279
	KPC-103	WP_231869650.1	**A281_ins(KDDKHSEAVIAA)_A282**	A281_ins (12)_A282
Other	KPC-107	WP_231869652.1	**V208_ins(SSPRAVTESLQKLTLGSALAAPQRQQFV)_D209**	V208_ins (28)_D209

^*a*^ Bold terms correspond to the proposed annotation.

^*b*^ “-” indicates the position in the sequence where the insertion takes place.

### KPC variants with two or more types of mutations

A significant subset of KPC variants (*n* = 57) exhibited multiple mutation types, with substitution-insertion (*n* = 30) and substitution-deletion (*n* = 20) combinations being the most prevalent. For annotation, we applied the same conventions previously established for individual substitutions, deletions, and insertions, separating multiple changes within a single variant with commas. Notably, we identified a distinct subgroup featuring contiguous substitutions and deletions that required special consideration.

Discrepancies emerged between MAFFT and structural alignment results for these variants, particularly in the localization of mutations. For instance, KPC-239 showed varying interpretations: MAFFT suggested G239_∆V_Y241D, while structural alignment indicated V240D_∆Y_G242. Nucleotide-level analysis revealed that these variants originate from out-of-frame deletions that simultaneously generate contiguous amino acid deletions and substitutions (Fig. 2G). This type of mutation change was observed in multiple KPC variants (Table 4).

**TABLE 4:** Annotation proposal for selected KPC variants containing contiguous substitution and deletion combination changes

Location	KPC variant	Accession number	Proposed annotation
Ω loop	KPC-92	WP_240067725.1	**L167_∆EL_N170D, H274Y**
	KPC-94	WP_219804981.1	**L169H_∆N_S171**
	KPC-115	WP_242934070.1	**E168_∆LN_S171P, H274Y**
	KPC-150	WP_328703060.1	**E166_∆LEL_N170H, H274Y**
	KPC-166	WP_279240783.1	**L169H_∆N_S171**
	KPC-225	WP_367187953.1	**I173_∆PGDAR_D179H**
237–243 loop	KPC-87	WP_213994593.1	**Y241_∆G_T243A**
	KPC-151	WP_268871856.1	**V240_∆YG_T243S**
	KPC-239	WP_394294905.1	**V240D_∆Y_G242**
Ω loop, 237–243 loop	KPC-155	WP_274293084.1	**L169P, Y241_∆G_T243A**
	KPC-185	WP_311033308.1	**D179G, Y241_∆G_T243A**

To resolve such ambiguities, we analyzed AlphaFold3 models. It is possible that X-ray crystallography would provide evidence about the localization of these structural changes; however, in the absence of such experimental data, we consider AlphaFold3 models a reliable alternative. Interestingly, some AlphaFold3-predicted structural positions (with pLDDT scores >80) diverged from structural alignment annotations, and in these cases, we prioritized the AlphaFold3 models for final annotation. For contiguous deletion-substitution events, we adopted a combined annotation format, with both types of changes separated by underscores, as previously described for deletions (Table 4). These findings highlight the necessity of integrating nucleotide alignments, structural alignments, and *in silico* modeling to accurately characterize complex residue alterations in KPC variants.

### Conclusion

**Fig 3:**
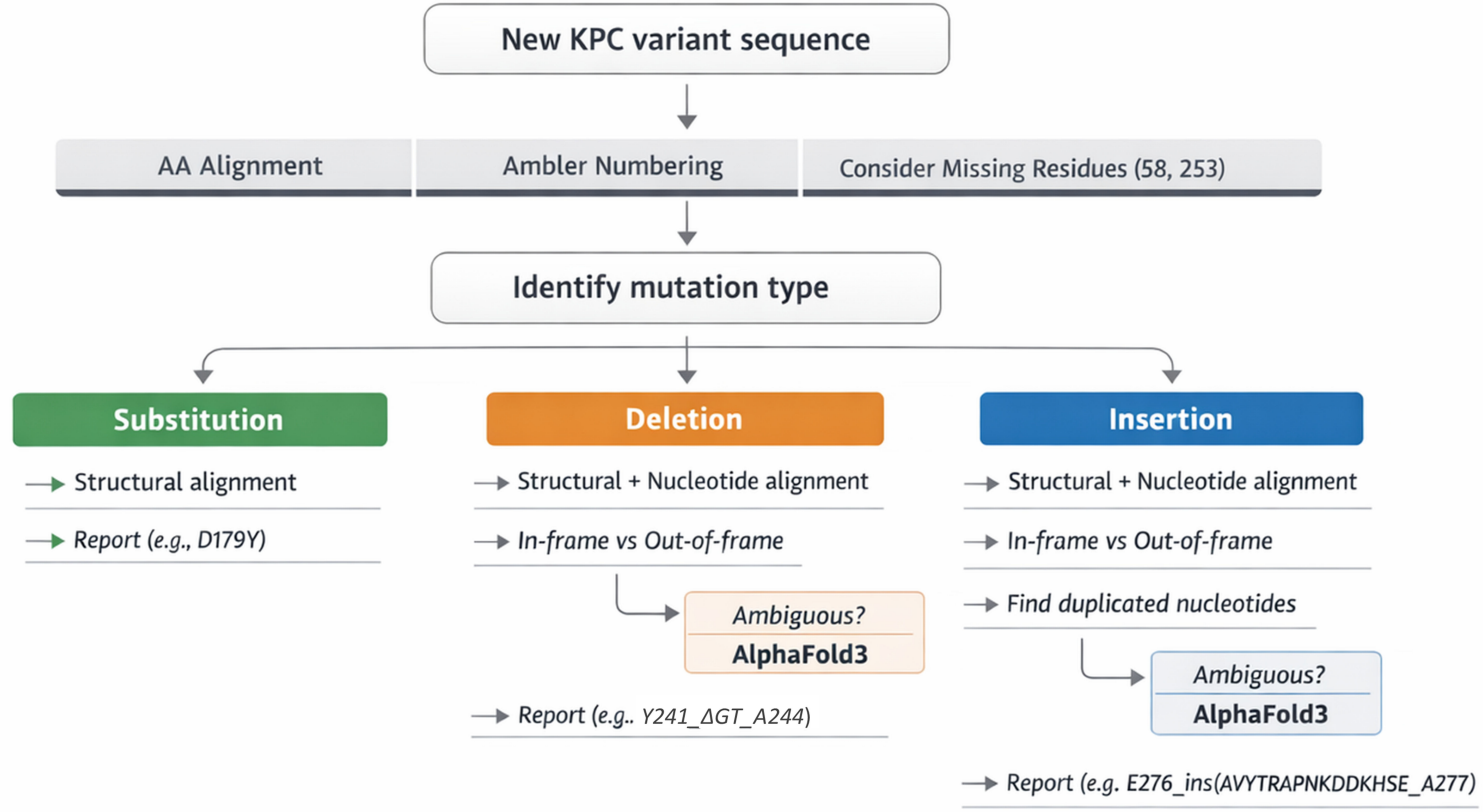
Flowchart summarizing the proposed steps to identify and correct the amino acid numbering in a new KPC variant. See text for details.

We describe a comprehensive revision of amino acid changes across the KPC β-lactamase family. It is our intent to provide specific guidelines to reach a consensus for numbering amino acid residues in KPC variants harboring substitutions, deletions, insertions, or a combination thereof. We presented the challenges encountered during our analysis and suggested conventions for special cases. A proposed flowchart is shown in Fig. 3.

We emphasize the critical importance of applying the Ambler numbering scheme, particularly noting the absence of residues 58 and 253 in KPC sequences, and strongly recommend analyzing each variant individually against KPC-2, taking into account structural changes rather than only using multiple-sequence alignments, which can bias results.

We evaluated different alignment algorithms and observed that relying on a single, currently available tool is insufficient, particularly for insertions and deletions. We therefore recommend combining amino acid-level structural alignments with nucleotide-level alignments to better understand the origin of each mutation, followed by manual curation to correct potential algorithmic errors.

In cases where alignment mapping remains ambiguous, we propose incorporating structural information using *in silico* models (e.g., AlphaFold3) to identify the biologically most plausible mutation locations. Although AlphaFold3 has not been formally validated for accurately predicting the structural impact of sequence perturbations and can poorly perform in highly dynamic regions and intrinsically disordered loops, it consistently achieves high accuracy for folded domains and provides reliable confidence metrics (pLDDT) (45–47). Consequently, in cases where sequence alignment is ambiguous, high-confidence AlphaFold models can provide valuable structural constraints that complement or exceed information available from sequence comparison alone. Furthermore, it has also been demonstrated that reproducibility and potential accuracy of AI-based predictions by AlphaFold3 still depend on the parameter settings used in the multi-sequence alignment, and even small differences in these settings can alter the outcome (47, 48). Finally, cross-validation with experimental results are essential to validate or refute AI-based predictions. We highlight the need for additional experimental data (e.g., by X-ray crystallography or NMR spectroscopy analyses) to validate particularly complex cases. Future efforts should focus on obtaining such experimental structures to complement computational predictions.

Given the emergence of multiple new KPC variants in the context of antimicrobial resistance and the approval of novel β-lactamase inhibitors targeting the KPC family, our systematic scheme aims to standardize the description of newly arising KPC variants and prevent discrepancies in future reports. To facilitate broader adoption of this proposal, particularly among users less familiar with β-lactamase structure and nomenclature, collaboration with database curators will be essential to support its practical implementation. A similar approach has been successfully implemented for class D β-lactamases through the SAND tool, which is freely available as a user-friendly Google Colab resource for the scientific community (49).

To align with the objectives of this study, the BLDB has already implemented the residue numbering system. Therefore, the substitutions, insertions, and deletions for KPC have been updated for compatibility with this numbering system and will be used going forward for any newly discovered KPC allele. The URL for this BLDB KPC sequence alignment addition is http://bldb.eu/alignment.php?align=A:KPC.

## MATERIALS AND METHODS

### Sequence database

In September 2025, we retrieved 267 sequences of the KPC family (including KPC-2) from the Beta-Lactamase DataBase (BLDB) (http://bldb.eu/) (21). Only variants with an associated nucleotide sequence deposited in the NCBI database were considered, while those with an “assigned status” were discarded.

### Sequence alignments

The amino acid sequences were aligned using the MAFFT algorithm (50), and residue changes relative to KPC-2 were identified using MSA2SNP (*https://github.com/pinbo/msa2snp*). Excel was then used to summarize the VCF output from MSA2SNP and create the initial table for number correction. To systematically analyze the distribution of amino acid changes across these variants, we classified the family into the following subgroups: (i) variants with only substitutions (*n* = 126); (ii) variants with only deletions (*n* = 17); (iii) variants with only insertions (*n* = 66); and (iv) variants with two or more types of amino acid changes, e.g., combined substitutions, deletions, or insertions (*n* = 57). Details on the composition of each subgroup are provided in the Supplemental Material.

In each subgroup, we confirmed amino acid changes through structural alignment against KPC-2 using the T-Coffee Expresso server v. 13.45.0.4846264 (51), including the KPC-2 crystal structure (PDB: 2OV5) as input in the “Structure(s) input” section, while the remaining settings were left as default. We also performed nucleotide alignment of variants against KPC-2 using the ClustalW algorithm (52) option included in the BioEdit v7.7.1.0 software. Alignments were visualized and manually curated using ESPript 3.2 (53) and BioEdit v7.7.1.0 (https://thalljiscience.github.io/). Due to the absence of amino acid residues at position 58 (between V57 and S59) and 253 (between P252 and T254) in the KPC-2 sequence, we entered “58 253” in the “Delete in seq numbering” section of the ESPript 3.2 server under Expert mode to harmonize the KPC-2 residue numbering with other class A β-lactamases.

### *In silico* modeling

We defined “complex cases” as those in which the alignment results obtained from nucleotide and structure-based algorithms disagreed regarding the location of changes compared to KPC-2 (e.g., a combination of substitutions and contiguous deletions, or deletions and insertions involving tandem residues). In these cases, *in silico* models were created using the AlphaFold3 server, and we analyzed the associated confidence metrics for each model (54).

We also cross-referenced all mutations with both literature reports in PubMed and crystal structures in the Protein Data Bank (PDB). Both searches were performed using each KPC variant as a query in both databases to verify whether our mutation descriptions matched previously available reports or deposited crystal structures. The proposed changed residue annotations for all KPC variants and AlphaFold3 metrics are provided in the Supplemental Material.

## SUPPLEMENTAL MATERIAL

Supplemental material aac.01868-25-s0001.pdf Supplemental figures and tables for Standardized numbering and alignment of the KPC family of β-lactamases.

ASM does not own the copyrights to Supplemental Material that may be linked to, or accessed through, an article. The authors have granted ASM a non-exclusive, world-wide license to publish the Supplemental Material files. Please contact the corresponding author directly for reuse.
