# Supplementary material for: Standardized numbering and alignment of the KPC family of β-lactamases
Source: Antimicrob Agents Chemother. 2026 May 29;70(7):e01868-25. doi: 10.1128/aac.01868-25 (PMC13321812; doi:10.1128/aac.01868-25)
Supplement: Supplemental material — Supplemental figures and tables for Standardized numbering and alignment of the KPC family of β-lactamases. [file aac.01868-25-s0001.pdf]

Supplemental Material A. Details of analyzed KPC variant subgroups

Type of AA change	Total	Derived from KPC-2	Derived from KPC-3
Only Substitutions	126	83	43
Only Deletions	17	13	4
Only Insertions	66	48	18
2 or more	57	39	18
Total	**266 (267 with KPC-2)**	183	83

a) Variants with Only Substitutions

Location of substitution	Total	Derived from KPC-2	Derived from KPC-3
Ω loop	47	31	16
237-243 loop	10	5	5
266-275 loop	4	2	2
Other	44	32	12
Combination of 2 or more spots	21	13	8
Total	**126**	83	43

b) Variants with Only Deletions

Location of Deletions	Total	Derived from KPC-2	Derived from KPC-3
Ω loop	11	8	3
237-243 loop	5	4	1
266-275 loop	-	-	-
Other	-	-	-
Combination of 2 or more spots	1	1	-
Total	**17**	13	4

c) Variants with Only Insertions

Location of Insertions	Total	Derived from KPC-2	Derived from KPC-3
Only Ω-loop	19	13	6
Only 237-243 loop	2	2	-
Only 266-275 loop	37	27	10
Other	-	-	-
Combination of 2 or more spots	8	6	2
Total	**66**	48	48

d) Variants with 2 or more types of AA changes

Type of AA change	Total	Derived from KPC-2	Derived from KPC-3
Ins-Del	6	3	3
Ins-Subs	30	24	6
Subs-Del	20	12	8
Subs-Del-Ins	1	-	1
Total	**57**	39	18

Accession #	KPC-2 or KPC-3	# of AA changes	Loop	Type AA change	S	D	I	AA change
WP_004199234.1_KPC-2	KPC-2	NA						
WP_004152396.1_KPC-3	KPC-3	1	266-275 loop	Substitution	S_1			H274Y
WP_015062847.1_KPC-4	KPC-2	2	237-243 loop , other location	Substitution	S_2			P104R, V240G
WP_015256839.1_KPC-5	KPC-2	1	other location	Substitution	S_1			P104R
WP_047665864.1_KPC-6	KPC-2	1	237-243 loop	Substitution	S_1			V240G
WP_020803043.1_KPC-7	KPC-3	2	other location	Substitution	S_2			M49I, H274Y
WP_063860797.1_KPC-8	KPC-3	2	237-243 loop	Substitution	S_2			V240G, H274Y
WP_063860633.1_KPC-10	KPC-3	2	other location	Substitution	S_2			P104R, H274Y
WP_032494844.1_KPC-11	KPC-2	1	other location	Substitution	S_1			P104L
WP_063860634.1_KPC-12	KPC-2	1	Omega loop	Substitution	S_1			L169M
WP_063860635.1_KPC-13	KPC-3	2	other location	Substitution	S_2			D92G, H274Y
WP_063860636.1_KPC-14	KPC-2	1	237-243 loop	Deletion		D_2		Y241_ΔGT_A244
WP_063860637.1_KPC-15	KPC-3	5	237-243 loop , other location	Substitution	S_5			P104R, A120L, G147K, V240G, H274Y
WP_063860638.1_KPC-16	KPC-2	2	other location	Substitution	S_2			P202S, F207L
WP_063860639.1_KPC-17	KPC-2	1	other location	Substitution	S_1			F207L
WP_063860640.1_KPC-18	KPC-2	1	other location	Substitution	S_1			V8I

WP_063860641.1_KPC-19	KPC-3	2	other location	Substitution	S_2			H274Y, N293T
ATM29810.1_KPC-20	KPC-2	1	other location	Substitution	S_1			V30I
WP_063860711.1_KPC-21	KPC-2	1	other location	Substitution	S_1			W105R
WP_063860723.1_KPC-22	KPC-2	2	other location	Substitution	S_2			W105G, F207L
WP_111672911.1_KPC-23	KPC-3	2	237-243 loop	Substitution	S_2			V240A, H274Y
WP_063860729.1_KPC-24	KPC-2	1	other location	Substitution	S_1			R6P
WP_065419571.1_KPC-25	KPC-2	1	Omega loop	Insertion			I_2	L167_ins(EL)_E168
WP_068981634.1_KPC-26	KPC-2	1	other location	Substitution	S_1			A18S
WP_077064886.1_KPC-27	KPC-3	2	other location	Substitution	S_2			W105R, H274Y
WP_072081992.1_KPC-28	KPC-3	2	237-243 loop	Deletion	S_1	D_2		Y241_ΔGT_A244, H274Y
WP_096807439.1_KPC-29	KPC-3	2	266-275 loop	Insertion	S_1		I_3	D272_ins(KDD)_K273, H274Y
WP_085562399.1_KPC-30	KPC-2	1	other location	Substitution	S_1			R6H
WP_073668892.1_KPC-31	KPC-3	2	Omega loop	Substitution	S_2			D179Y, H274Y
WP_073800284.1_KPC-32	KPC-3	3	Omega loop, 237-243 loop	Substitution	S_3			D179Y, T243M, H274Y
WP_101140102.1_KPC-33	KPC-2	1	Omega loop	Substitution	S_1			D179Y
WP_109545044.1_KPC-34	KPC-2	1	266-275 loop	Insertion			I_8	A277_ins(KDDKHSEA)_V278
WP_111273852.1_KPC-35	KPC-2	1	Omega loop	Substitution	S_1			L169P

WP_114699267.1_KPC-36	KPC-3	2	Omega loop	Substitution	S_2			D163E, H274Y
WP_116786832.1_KPC-37	KPC-2	2	Omega loop, other location	Substitution	S_2			W165R, F207L
WP_123002101.1_KPC-38	KPC-3	2	other location	Substitution	S_2			H274Y, V292A
WP_128268237.1_KPC-39	KPC-3	2	Omega loop	Substitution	S_2			A172T, H274Y
WP_115470049.1_KPC-40	KPC-3	3	Omega loop, 237-243 loop	Insertion, Substitution	S_2		I_2	L167_ins(EL)_E168, T243S, H274Y
WP_148044419.1_KPC-41	KPC-3	2	266-275 loop	Insertion	S_1		I_3	K270_ins(PNK)_D271, H274Y
WP_136512070.1_KPC-42	KPC-2	1	other location	Substitution	S_1			T254A
WP_136512071.1_KPC-43	KPC-2	1	other location	Substitution	S_1			Q191R
WP_140423311.1_KPC-44	KPC-2	1	266-275 loop	Insertion			I_15	E276_ins(AVYTRAPNKDDKHSE)_A277
WP_148044420.1_KPC-45	KPC-2	1	other location	Substitution	S_1			T93K
WP_148044421.1_KPC-46	KPC-3	2	Omega loop	Substitution	S_2			L169P, H274Y
WP_213994588.1_KPC-47	KPC-3	3	Omega loop, 237-243 loop	Substitution	S_3			A172T, T243A, H274Y
WP_213994589.1_KPC-48	KPC-3	3	Omega loop	Substitution	S_3			L169P, A172T, H274Y
WP_197749402.1_KPC-49	KPC-3	2	Omega loop	Substitution	S_2			R164S, H274Y
WP_171476788.1_KPC-50	KPC-3	2	266-275 loop	Insertion	S_1		I_3	H274Y, V278_ins(EAV)_I279
WP_158208923.1_KPC-51	KPC-2	3	Omega loop, 237-243 loop, 266-275 loop	Substitution	S_3			D179N, Y241H, H274N

WP_158208806.1_KPC-52	KPC-2	2	266-275 loop , Omega loop	Substitution, Insertion	S_1		I_1	D179Y, V263_ins(V)_Y264
WP_156649232.1_KPC-53	KPC-3	2	Omega loop	Insertion	S_1		I_2	L167_ins(EL)_E168, H274Y
WP_160164839.1_KPC-54	KPC-2	1	other location	Substitution	S_1			A62S
WP_168247883.1_KPC-55	KPC-2	1	other location	Substitution	S_1			Y264N
WP_087744800.1_KPC-56	KPC-3	2	other location	Substitution	S_2			H274Y, G294W
WP_171476789.1_KPC-57	KPC-2	1	Omega loop	Substitution	S_1			D179V
WP_179284320.1_KPC-58	KPC-2	1	266-275 loop	Insertion			I_8	D272_ins(NRAPNKDD)_K273
WP_179284322.1_KPC-59	KPC-2	1	other location	Substitution	S_1			G89D
WP_179284324.1_KPC-60	KPC-2	1	other location	Substitution	S_1			A120T
WP_179284328.1_KPC-61	KPC-3	2	Omega loop	Substitution	S_2			S171P, H274Y
WP_204376229.1_KPC-62	KPC-3	2	Omega loop	Substitution	S_2			L169Q, H274Y
WP_204376230.1_KPC-63	KPC-3	2	237-243 loop	Substitution	S_2			Y241S, H274Y
WP_204376231.1_KPC-64	KPC-3	4	237-243 loop , 266-275 loop , Omega loop	Insertion, Substitution	S_3		I_1	S181_ins(S)_S182, T243A, Y264H, H274Y
WP_204376232.1_KPC-65	KPC-3	2	Omega loop	Insertion	S_1		I_2	T180_ins(YT)_S181, H274Y
WP_188331871.1_KPC-66	KPC-3	2	Omega loop	Deletion	S_1	D_2		L167_ΔEL_N170, H274Y
WP_210204487.1_KPC-67	KPC-3	2	266-275 loop	Insertion	S_1		I_6	D272_ins(KDDKDD)_K273, H274Y

WP_210204468.1_KPC-68	KPC-3	2	Omega loop	Insertion	S_1		I_2	S182_inss(SS)_P183, H274Y
WP_210205486.1_KPC-69	KPC-3	2	Omega loop	Insertion	S_1		I_2	W165_ins(GL)_E166, H274Y
WP_210205477.1_KPC-70	KPC-3	3	Omega loop, 266-275 loop	Substitution	S_3			D179Y, T265A, H274Y
WP_194293134.1_KPC-71	KPC-2	1	Omega loop	Insertion			I_1	S181_ins(S)_S182
WP_188331872.1_KPC-72	KPC-2	1	Omega loop	Substitution	S_1			A172D
WP_188331873.1_KPC-73	KPC-2	2	Omega loop, 266-275 loop	Insertion, Deletion		D_2	I_6	L167_ΔEL_N170, S275_ins(KDDKHS)_E276
WP_188331874.1_KPC-74	KPC-2	1	237-243 loop	Deletion		D_2		C238_ΔGV_Y241
WP_188331875.1_KPC-75	KPC-2	1	other location	Substitution	S_1			L13F
WP_194293135.1_KPC-76	KPC-2	2	Omega loop, 266-275 loop	Substitution, Insertion	S_1		I_7	D179Y, N269_ins(VYTRAPN)_K270
WP_194293136.1_KPC-77	KPC-2	1	Omega loop	Substitution	S_1			R164P
WP_197749403.1_KPC-78	KPC-2	1	Omega loop	Substitution	S_1			D179A
WP_197749404.1_KPC-79	KPC-2	1	266-275 loop	Insertion			I_7	N269_ins(VYTRAPN)_K270
WP_204376233.1_KPC-80	KPC-2	1	266-275 loop	Insertion			I_3	K270_ins(PNK)_D271
WP_204376234.1_KPC-81	KPC-2	1	Omega loop	Deletion		D_1		A172_ΔI_P174
WP_202781289.1_KPC-82	KPC-2	1	266-275 loop	Insertion			I_2	S275_ins(DS)_E276
WP_240067723.1_KPC-83	KPC-2	1	other location	Substitution	S_1			A34T
WP_213994590.1_KPC-84	KPC-2	1	237-243 loop	Substitution	S_1			T243P

WP_213994591.1_KPC-85	KPC-3	2	Omega loop	Substitution	S_2			A172V, H274Y
WP_213994592.1_KPC-86	KPC-2	1	Omega loop	Substitution	S_1			D179G
WP_213994593.1_KPC-87	KPC-2	2	237-243 loop	Substitution, Deletion	S_1	D_1		Y241_ΔG_T243A
WP_213994594.1_KPC-88	KPC-2	1	Omega loop	Substitution	S_1			D176Y
WP_240067724.1_KPC-89	KPC-3	2	237-243 loop	Substitution	S_2			T243M, H274Y
WP_219833567.1_KPC-90	KPC-2	1	Omega loop	Insertion			I_2	T180_ins(YT)_S181
WP_219860720.1_KPC-91	KPC-3	2	other location	Substitution	S_2			V103L, H274Y
WP_240067725.1_KPC-92	KPC-3	3	Omega loop	Substitution, Deletion	S_2	D_2		L167_ΔEL_N170D, H274Y
WP_243939160.1_KPC-93	KPC-2	1	266-275 loop	Insertion			I_5	N269_ins(NRAPN)_K270
WP_219804981.1_KPC-94	KPC-3	3	Omega loop	Substitution, Deletion	S_2	D_1		L169H_ΔN_S171, H274Y
WP_219795346.1_KPC-95	KPC-3	3	Omega loop	Substitution	S_3			A172T, D179Y, H274Y
WP_231869639.1_KPC-96	KPC-2	1	237-243 loop	Substitution	S_1			Y241N
WP_231869640.1_KPC-97	KPC-2	1	266-275 loop	Insertion			I_5	V278_ins(NSEAV)_I279
WP_231869634.1_KPC-98	KPC-3	2	Omega loop	Substitution	S_2			R164H, H274Y
WP_262697140.1_KPC-99	KPC-2	1	Omega loop	Substitution	S_1			R164S
WP_250200322.1_KPC-100	KPC-2	1	237-243 loop	Substitution	S_1			T243M
WP_262697129.1_KPC-101	KPC-2	1	266-275 loop	Insertion			I_2	H274_ins(KH)_S275

WP_231873781.1_KPC-102	KPC-2	2	Omega loop, 237-243 loop	Substitution	S_2			D179Y, Y241D
WP_231869650.1_KPC-103	KPC-2	1	266-275 loop	Insertion			I_12	A281_ins(KDDKHSEAVIAA)_A282
WP_231869651.1_KPC-104	KPC-2	2	Omega loop, 266-275 loop	Insertion			I_2, I_6	T180_ins(YT)_S181, E276_ins(DDKHSE)_A277
WP_231869653.1_KPC-105	KPC-2	2	Omega loop, 266-275 loop	Substitution, Insertion	S_1		I_15	L169Q, E276_ins(AVYTRAPNKDDKHSE)_A277
WP_231869655.1_KPC-106	KPC-2	2	Omega loop, 266-275 loop	Insertion			I_2, I_4	T180_ins(YT)_S181, V278_ins(SEAV)_I279
WP_231869652.1_KPC-107	KPC-2	1	other location	Insertion			I_27	V208_ins (SSPRAVTESLQKLTLGSALAAPQRQQFV)_D209
WP_231869654.1_KPC-108	KPC-2	2	Omega loop, 266-275 loop	Insertion			I_10, I_15	E188_ins(NTSSPRAVTE)_S189, E276_ins (AVYTRAPNKDDKHSE)_A277
WP_274293083.1_KPC-109	KPC-2	1	266-275 loop	Insertion			I_6	H274_ins(NKDDKY)_S275
WP_257394580.1_KPC-110	KPC-3	3	Omega loop, other location	Substitution	S_3			G42R, D179Y, H274Y
WP_257394581.1_KPC-111	KPC-3	3	Omega loop	Substitution	S_3			P174L, D179Y, H274Y
WP_240067722.1_KPC-112	KPC-2	2	Omega loop, 237-243 loop	Deletion		D_2, D_2		L167_ΔEL_N170, Y241_ΔGT_A244
WP_242934068.1_KPC-113	KPC-2	1	266-275 loop	Insertion			I_1	R266_ins(G)_A267
WP_242934069.1_KPC-114	KPC-2	1	Omega loop	Insertion			I_2	S182_ins(SS)_P183
WP_242934070.1_KPC-115	KPC-3	3	Omega loop	Deletion, Substitution	S_2	D_2		E168_ΔLN_S171P, H274Y
WP_242934071.1_KPC-116	KPC-2	1	Omega loop	Substitution	S_1			S171F
WP_242934072.1_KPC-117	KPC-2	4	other location	Insertion, Substitution	S_3		I_3, I_7	S2_ins(RCP)_L3I, Y4F, R6_ins(F)_L7
WP_242934073.1_KPC-118	KPC-3	3	Omega loop, other location	Substitution	S_3			V103L, W165R, H274Y

WP_242934074.1_KPC-119	KPC-2	1	other location	Substitution	S_1			Q38H
WP_242934075.1_KPC-120	KPC-2	2	Omega loop, other location	Substitution	S_2			P104R, D179Y
WP_242934076.1_KPC-121	KPC-3	2	Omega loop	Insertion	S_1		I_1	S181_ins(S)_S182, H274Y
WP_242934077.1_KPC-122	KPC-3	2	other location	Substitution	S_2			V8I, H274Y
WP_242934078.1_KPC-123	KPC-2	2	Omega loop, 266-275 loop	Insertion			I_2, I_7	T180_ins(YT)_S181, A277_ins(DDKHSEA)_V278
WP_290468351.1_KPC-124	KPC-3	3	Omega loop	Substitution, Deletion	S_2	D_2		L167_ΔEL_N170, D179Y, H274Y
WP_248496376.1_KPC-125	KPC-3	2	Omega loop	Substitution	S_2			D179A, H274Y
WP_249828063.1_KPC-126	KPC-2	1	Omega loop	Substitution	S_1			A172V
WP_250200323.1_KPC-127	KPC-2	2	Omega loop	Insertion, Substitution	S_1		I_2	L167_ins(EL)_E168, A172T
WP_250200324.1_KPC-128	KPC-2	2	Omega loop, 237-243 loop	Substitution	S_2			D179Y, T243M
WP_290468352.1_KPC-129	KPC-2	2	Omega loop, 266-275 loop	Substitution, Insertion	S_1		I_8	N170H, K270_ins(VYTRAPNK)_D271
WP_253892094.1_KPC-130	KPC-3	2	Omega loop	Substitution	S_2			D179G, H274Y
WP_256875604.1_KPC-131	KPC-2	1	other location	Substitution	S_1			T216A
WP_257102137.1_KPC-132	KPC-3	2	266-275 loop	Insertion	S_1		I_9	K273_ins(SRAPNKDDK)_H274Y
WP_257102138.1_KPC-133	KPC-2	2	Omega loop, 266-275 loop	Substitution, Insertion	S_1		I_15	D179G, E276_ins(AVYTRAPNKDDKHSE)_A277
WP_262697130.1_KPC-134	KPC-2	2	Omega loop, 266-275 loop	Substitution, Insertion	S_1		I_8	D179A, D272_ins(NRAPNKDD)_K273
WP_262697131.1_KPC-135	KPC-2	2	Omega loop, 266-275 loop	Deletion, Insertion		D_2	I_15	L167_ΔEL_N170, E276_ins (AVYTRAPNKDDKHSE)_A277

WP_279240775.1_KPC-136	KPC-2	1	Omega loop	Substitution	S_1			P174L
WP_318245547.1_KPC-137	KPC-2	2	Omega loop	Substitution	S_2			P174L, G175D
WP_262697133.1_KPC-138	KPC-2	3	Omega loop, 237-243 loop , 266-275 loop	Substitution, Insertion	S_2		I_5	L169P, T243A, V278_ins(NSEAV)_I279
WP_262697134.1_KPC-139	KPC-2	2	Omega loop, 266-275 loop	Substitution, Insertion	S_1		I_6	D179Y, S275_ins(KDDKHS)_E276
WP_262697135.1_KPC-140	KPC-2	2	Omega loop, 266-275 loop	Substitution, Insertion	S_1		I_15	D179N, E276_ins(AVYTRAPNKDDKHSE)_A277
WP_262697136.1_KPC-141	KPC-2	2	Omega loop, 237-243 loop	Substitution, Insertion	S_1		I_1	D179Y, G239_ins(G)_V240
WP_262697137.1_KPC-142	KPC-2	2	Omega loop, 266-275 loop	Insertion			I_1, I_6	S181_ins(S)_S182, V278_ins(KHSEAV)_I279
WP_262697138.1_KPC-143	KPC-2	2	Omega loop, other location	Substitution	S_2			D179Y, T187S
WP_262697139.1_KPC-144	KPC-2	1	Omega loop	Substitution	S_1			A172T
WP_268871853.1_KPC-145	KPC-2	2	Omega loop, 266-275 loop	Substitution	S_2			D179Y, T265A
WP_268871854.1_KPC-146	KPC-2	1	other location	Substitution	S_1			L3M
WP_268871855.1_KPC-147	KPC-2	1	other location	Substitution	S_1			L9M
WP_264254632.1_KPC-148	KPC-3	2	266-275 loop	Insertion	S_1		I_15	H274Y, E276_ins(AVYTRAPNKDDKYSE)_A277
WP_328703059.1_KPC-149	KPC-3	2	other location	Substitution	S_2			N214S, H274Y
WP_328703060.1_KPC-150	KPC-3	3	Omega loop	Substitution, Deletion	S_2	D_3		E166_ΔLEL_N170H, H274Y
WP_268871856.1_KPC-151	KPC-2	2	237-243 loop	Substitution, Deletion	S_1	D_2		V240_ΔYG_T243S
WP_328703061.1_KPC-152	KPC-3	3	Omega loop, 266-275 loop	Insertion	S_1		I_2, I_10	L167_ins(EL)_E168, H274Y_ins(TRAPNKDDKY)_S275

WP_268871857.1_KPC-153	KPC-3	3	Omega loop, 266-275 loop	Insertion, Substitution	S_2		I_2	L167_ins(EL)_E168, H274Y, E276D
WP_309367897.1_KPC-154	KPC-3	2	266-275 loop	Insertion	S_1		I_10	H274Y, S275_ins(RAPNKDDKYS)_E276
WP_274293084.1_KPC-155	KPC-2	3	Omega loop, 237-243 loop	Substitution, Deletion	S_2	D_1		L169P, Y241_ΔG_T243A
WP_274293085.1_KPC-156	KPC-2	1	other location	Substitution	S_1			G291D
WP_259115967.1_KPC-157	KPC-2	1	other location	Substitution	S_1			N132S
WP_304487000.1_KPC-158	KPC-2	1	237-243 loop	Substitution	S_1			A244V
WP_279240776.1_KPC-159	KPC-2	2	Omega loop, 237-243 loop	Substitution, Deletion	S_1	D_2		D179E, Y241_ΔGT_A244
WP_279240777.1_KPC-160	KPC-2	1	Omega loop	Deletion		D_2		L167_ΔEL_N170
WP_279240778.1_KPC-161	KPC-2	1	266-275 loop	Insertion			I_11	A281_ins(DDKHSEAVIAA)_A282
WP_279240779.1_KPC-162	KPC-2	1	266-275 loop	Insertion			I_7	D271_ins(TRAPNKD)_D272
WP_279240780.1_KPC-163	KPC-2	1	266-275 loop	Insertion			I_6	S275_ins(KDDKHS)_E276
WP_279240781.1_KPC-164	KPC-2	1	266-275 loop	Insertion			I_3	V278_ins(EAV)_I179
WP_279240782.1_KPC-165	KPC-2	1	Omega loop	Substitution	S_1			N170D
WP_279240783.1_KPC-166	KPC-2	2	Omega loop	Substitution, Deletion	S_1	D_1		L169H_ΔN_S171
WP_279240784.1_KPC-167	KPC-3	3	Omega loop, 266-275 loop	Substitution, Insertion	S_2		I_6	D179Y, H274Y, E276_ins(DDKYSE)_A277
WP_318245548.1_KPC-168	KPC-2	1	Omega loop	Substitution	S_1			D176N
WP_338424102.1_KPC-169	KPC-2	1	Omega loop	Deletion		D_3		G175_ΔDAR_D179

WP_311033307.1_KPC-170	KPC-2	1	Omega loop	Substitution	S_1			D179N
WP_338424103.1_KPC-171	KPC-2	1	266-275 loop	Insertion			I_8	A281_ins(DSEAVIAA)_A282
WP_338424104.1_KPC-172	KPC-2	1	Omega loop	Substitution	S_1			D179E
WP_338424105.1_KPC-173	KPC-2	1	Omega loop	Insertion			I_2	L169_ins(KL)_N170
WP_338424106.1_KPC-174	KPC-2	1	237-243 loop	Deletion		D_1		V240_ΔY_G242
WP_338424107.1_KPC-175	KPC-2	1	Omega loop	Insertion			I_1	T180_ins(T)_S181
WP_318245549.1_KPC-176	KPC-2	1	Omega loop	Substitution	S_1			S171P
WP_338424108.1_KPC-177	KPC-2	1	Omega loop	Insertion			I_13	Q191_ins(NTSSPRAVTESLQ)_K192
WP_290468353.1_KPC-178	KPC-3	2	Omega loop	Substitution	S_2			P174L, H274Y
WP_290468354.1_KPC-179	KPC-2	2	Omega loop, other location	Substitution, Insertion	S_1		I_1	A133T, S181_ins(S)_S182
WP_290468355.1_KPC-180	KPC-2	1	Omega loop	Substitution	S_1			R164H
WP_163591930.1_KPC-181	KPC-2	1	266-275 loop	Substitution	S_1			E276D
WP_304487001.1_KPC-182	KPC-2	1	other location	Substitution	S_1			L287I
WP_304487002.1_KPC-183	KPC-3	2	266-275 loop	Insertion	S_1		I_6	H274Y, S275_ins(KDDKYS)_E276
WP_304487003.1_KPC-184	KPC-3	2	266-275 loop	Substitution	S_2			H274Y, E276D
WP_311033308.1_KPC-185	KPC-2	3	Omega loop, 237-243 loop	Substitution, Deletion	S_2	D_1		D179G, Y241_ΔG_T243A
WP_311033309.1_KPC-186	KPC-2	1	Omega loop	Deletion		D_2		A177_ΔRD_T180

WP_311033310.1_KPC-187	KPC-2	2	Omega loop	Substitution	S_2			R164H, D179Y
WP_375516059.1_KPC-188	KPC-2	2	Omega loop, 266-275 loop	Insertion			I_1, I_7	S181_ins(S)_S182, N269_ins(VYTRAPN)_K270
WP_311033311.1_KPC-189	KPC-2	2	Omega loop	Substitution	S_2			A172T, D179Y
WP_311033312.1_KPC-190	KPC-2	2	Omega loop, 237-243 loop	Substitution	S_2			D179Y, A244V
WP_311033313.1_KPC-191	KPC-2	2	Omega loop	Substitution, Deletion	S_1	D_1		A177_ΔR_D179P
WP_311033314.1_KPC-192	KPC-2	1	266-275 loop	Insertion			I_10	N269_ins(VLAVYTRAPN)_K270
WP_318245550.1_KPC-193	KPC-2	1	266-275 loop	Insertion			I_3	K270_ins(ANK)_D271
WP_318245551.1_KPC-194	KPC-2	2	Omega loop	Substitution	S_2			D179Y, P183L
WP_318245552.1_KPC-195	KPC-2	1	other location	Substitution	S_1			E288D
WP_318245553.1_KPC-196	KPC-2	2	Omega loop	Substitution, Insertion	S_1		I_2	L167_ins(EL)_E168, D179Y
WP_316472302.1_KPC-197	KPC-3	4	Omega loop, 266-275 loop	Substitution, Insertion, Deletion	S_2	D_2	I_2	L167_ΔEL_N170, A177E, H274Y, S275_ins(DS)_E276
WP_305449896.1_KPC-201	KPC-3	2	Omega loop	Deletion	S_1	D_3		R161_ΔLDR_W165, H274Y
WP_328703062.1_KPC-202	KPC-2	1	266-275 loop	Insertion			I_10	A283_ins(NSEAVIAAAA)_R284
WP_321140136.1_KPC-203	KPC-3	3	Omega loop, 266-275 loop	Insertion, Deletion	S_1	D_2	I_9	L167_ΔEL_N170, P268_ins(MLAVYTRAP)_N269, H274Y
WP_265432541.1_KPC-204	KPC-2	1	266-275 loop	Insertion			I_3	K273_ins(DDK)_H274
WP_322972495.1_KPC-205	KPC-3	2	266-275 loop	Insertion	S_1		I_5	N269_ins(NRAPN)_K270, H274Y
WP_328703063.1_KPC-206	KPC-2	1	Omega loop	Insertion			I_2	T180_insYS_S181

WP_328703064.1_KPC-207	KPC-2	2	Omega loop, 266-275 loop	Substitution, Insertion	S_1		I_1	L169Q, S275_ins(S)_E276
WP_328703065.1_KPC-208	KPC-2	2	Omega loop	Substitution	S_2			L169Q, D179Y
WP_338424109.1_KPC-209	KPC-2	1	Omega loop	Insertion			I_6	A185_insTSSPRA_V186
WP_336433394.1_KPC-211	KPC-2	1	Omega loop	Deletion		D_4		G175_ΔDARD_T180
WP_336434105.1_KPC-212	KPC-2	1	Omega loop	Substitution	S_1			R164L
WP_336464807.1_KPC-213	KPC-2	1	Omega loop	Substitution	S_1			D163G
WP_336433450.1_KPC-214	KPC-2	1	Omega loop	Substitution	S_1			R178H
WP_336433517.1_KPC-215	KPC-2	1	Omega loop	Substitution	S_1			I173S
WP_338424110.1_KPC-216	KPC-3	2	Omega loop	Insertion	S_1		I_1	L169_ins(K)_N170, H274Y
WP_367187949.1_KPC-217	KPC-3	4	Omega loop, 266-275 loop, other location	Substitution	S_4			A172D, H274Y, E276D, N293T
WP_367187950.1_KPC-218	KPC-3	4	Omega loop, 266-275 loop, other location	Substitution	S_4			P174L, H274Y, E276D, N293T
WP_367187951.1_KPC-223	KPC-2	1	other location	Substitution	S_1			V250F
WP_367187952.1_KPC-224	KPC-2	1	Omega loop	Substitution	S_1			L169Q
WP_367187953.1_KPC-225	KPC-3	3	Omega loop	Deletion, Substitution	S_2	D_5		I173_ΔPGDAR_D179H, H274Y
WP_367187954.1_KPC-226	KPC-2	1	237-243 loop	Deletion		D_2		V240_ΔYG_T243
WP_367187955.1_KPC-227	KPC-2	2	Omega loop, 266-275 loop	Substitution, Insertion	S_1		I_3	D179Y, K273_ins(DDK)_H274

WP_348866574.1_KPC-228	KPC-2	1	Omega loop	Deletion		D_4		L167_ΔELNS_A172
WP_367187956.1_KPC-230	KPC-2	2	Omega loop, 266-275 loop	Substitution, Insertion	S_1		I_6	R164S, S275_ins(KDDKHS)_E276
WP_375516060.1_KPC-231	KPC-3	2	Omega loop	Substitution	S_2			S171Y, H274Y
WP_375516061.1_KPC-232	KPC-3	2	other location	Substitution	S_2			T93K, H274Y
WP_375516062.1_KPC-233	KPC-2	1	266-275 loop	Insertion			I_6	P268_ins(IYTRAP)_N269
WP_376797688.1_KPC-234	KPC-2	2	Omega loop, 237-243 loop	Substitution	S_2			D179G, Y241D
WP_394294902.1_KPC-236	KPC-2	1	other location	Substitution	S_1			Q87R
WP_394294903.1_KPC-237	KPC-2	1	Omega loop	Deletion		D_1		N170_ΔS_A172
WP_394294904.1_KPC-238	KPC-3	3	Omega loop, 237-243 loop	Substitution, Deletion	S_2	D_2		L167_ΔEL_N170, V240E, H274Y
WP_394294905.1_KPC-239	KPC-3	3	237-243 loop	Substitution, Deletion	S_2	D_1		V240D_ΔY_G242, H274Y
WP_394294906.1_KPC-240	KPC-3	2	Omega loop	Deletion	S_1	D_4		E166_ΔLELN_S171, H274Y
WP_394294907.1_KPC-241	KPC-2	1	Omega loop	Deletion		D_3		L167_ΔELN_S171
WP_394294908.1_KPC-242	KPC-2	2	Omega loop, 266-275 loop	Substitution, Insertion	S_1		I_9	D179Y, N269_ins(LAVYTRAPN)_K270
WP_408906771.1_ KPC_243	KPC-2	1	266-275 loop	Insertion			I_4	N269_ins(RAPN)_K270
WP_408906772.1_ KPC_244	KPC-2	1	other location	Substitution, Insertion	S_1			N214T
WP_405259240.1_KPC_245	KPC-3	3	266-275 loop	Substitution, Insertion	S_2		I_9	D272_ins(YTRAPNKDD)_K273, H274Y, E276D
WP_397422731.1_KPC-246	KPC-2	2	other location, Omega loop	Substitution	S_2			P104R, D179V

WP_397411417.1_KPC-247	KPC-2	2	other location, 266-275 loop	Substitution, Insertion	S_1		I_6	P104R, N269_ins(YTRAPN)_K270
WP_396604145.1_KPC-248	KPC-3	2	other location	Substitution	S_2			K73R, H274Y
WP_420354097.1_KPC-249	KPC-2	1	Omega loop	Insertion			I_2	S181_ins(TS)_S182
WP_407066349.1_KPC-250	KPC-2	2	Omega loop, 266-275 loop	Substitution, Insertion	S_1		I_1	D179Y, N269_ins(N)_K270
WP_420354098.1_KPC-251	KPC-3	3	Omega loop, 266-275 loop	Insertion	S_1		I_1, I_15	S181_ins(S)_S182, H274Y, E276_ins (AVYTRAPNKDDKYSE)_A277
WP_416045607.1_KPC-252	KPC-2	1	237-243 loop	Insertion			I_6	A248_ins(AANDYA)_V249
WP_420354099.1_KPC-255	KPC-2	2	Omega loop, 266-275 loop	Substitution	S_2			D179Y, P268H
WP_420354100.1_KPC-256	KPC-2	2	Omega loop, 266-275 loop	Substitution, Insertion	S_1		I_15	D179Y, E276_ins(AVYTRAPNKDDKYSE) _A277
WP_420354101.1_KPC-257	KPC-2	2	Omega loop, 266-275 loop	Deletion, Insertion		D_2	I_8	L167_ΔEL_N170, K270_ins(VYTRAPNK) _D271
WP_420354102.1_KPC-258	KPC-2	3	Omega loop	Substitution, Deletion	S_2		I_13	R161_ΔLDRWELELNSAIP_G175H, D176G
WP_191678214.1_KPC-259	KPC-2	1	266-275 loop	Insertion			I_5	N269_ins(TRAPN)_K270
WP_420354103.1_KPC-260	KPC-3	3	Omega loop, 266-275 loop	Deletion, Insertion	S_1	D_2	I_2	L167_ΔEL_N170, H274Y, S275_ins(DS) _E276
WP_285228448.1_KPC-261	KPC-2	2	Omega loop, 266-275 loop	Substitution, Insertion	S_1		I_6	D179Y, D271_ins(EAPNKD)_D272
WP_415968006.1_KPC-262	KPC-2	1	266-275 loop	Substitution	S_1			P268S
WP_420354104.1_KPC-263	KPC-2	1	237-243 loop	Insertion			I_1	G239_ins(G)_V240
WP_346724470.1_KPC-264	KPC-2	1	other location	Substitution	S_1			S109P
WP_430673481.1_KPC-265	KPC-2	1	266-275 loop	Insertion			I_15	I279_ins(TRAPNKDDKHSEAVI)_A280

WP_430673482.1_KPC-266	KPC-2	1	266-275 loop	Insertion			I_6	A281_ins(EAVIAA)_A282
WP_430673483.1_KPC-267	KPC-3	3	Omega loop, 266-275 loop	Substitution, Insertion	S_2		I_3	D179N, H274Y, E276_ins(YSE)_A277
WP_430673484.1_KPC-268	KPC-2	2	Omega loop, 237-243 loop	Substitution, Deletion	S_1	D_2		D179Y, Y241_ΔGT_A244
WP_430673485.1_KPC-269	KPC-2	2	Omega loop, 237-243 loop	Substitution, Deletion	S_1	D_2		D179H, Y241_ΔGT_A244
WP_430673486.1_KPC-270	KPC-3	2	266-275 loop	Insertion	S_1		I_19	H274Y, A283_ins (TRAPNKDDKYSEAVIAAAA)_R284
WP_430673487.1_KPC-271	KPC-2	2	Omega loop, 266-275 loop	Substitution, Insertion	S_1		I_5	D179Y, H274_ins(KDDKH)_S275
WP_430673488.1_KPC-272	KPC-3	3	Omega loop, 266-275 loop	Deletion, Insertion	S_1		I_2, I_5	L167_ins(EL)_E168, H274Y, V278_ins (NSEAV)_I279
WP_430673489.1_KPC-273	KPC-2	1	Omega loop	Insertion			I_2	D176_ins(GD)_A177
WP_430673490.1_KPC-274	KPC-2	1	Omega loop	Substitution	S_1			D179H
WP_430673491.1_KPC-275	KPC-2	2	Omega loop, 237-243 loop	Substitution	S_2			D179Y, T243P
WP_073693425.1_KPC-276	KPC-3	2	237-243 loop	Substitution	S_2			T243A, H274Y
WP_435892086.1_KPC-277	KPC-2	1	Omega loop	Insertion			I_1	D179_ins(N)_T180
WP_435892087.1_KPC-278	KPC-2	1	266-275 loop	Insertion			I_6	D271_ins(RAPNKD)_D272
WP_435892088.1_KPC-279	KPC-2	3	Omega loop, 237-243 loop	Deletion, Substitution	S_1	D_2, D_1		L167_ΔEL_N170, Y241_ΔG_T243A
WP_435892089.1_KPC-280	KPC-2	2	237-243 loop	Substitution, Deletion	S_1	D_1		G239_ΔV_Y241D
WP_435892090.1_KPC-281	KPC-2	1	other location	Substitution	S_1			A257S

Analysis AlphaFold 3 metrics

Metric	Model or residue level	Description	Assessment
**pLDDT**	residue	confidence of residue modelling	0–100<br>>90 — sidechains correct<br>>70 — backbone is correct<br><50 — probably unstructured
**PAE**	residue	confidence in distance between two residues	0–30 Å (lower → more confident)
**pTM**	model	confidence of protein fold	0–1<br>>0.7–0.9 — chance of being the correct fold<br>>0.5 — likely the correct fold<br><0.2 — unlikely to be correctly folded

Adapted from Rennie ML, Oliver MR. 2025. Emerging frontiers in protein structure prediction following the AlphaFold revolution. J R Soc Interface 22.

KPC-66

Model 0 was selected

AlphaFold model	pTM	pLDDT_global	pLDDT_region_ 164-170	RMSD vs model 0 (global)	RMSD vs model 0 (region 164-170)
Model 0	0.9	89.74	77.68	-	-
Model 1	0.9	89.57	77.23	0.085	0.650
Model 2	0.89	89.62	77.20	0.072	0.574
Model 3	0.89	89.65	77.62	0.053	0.099
Model 4	0.89	89.44	77.15	0.103	2.996

Models colored based on pLDDT scores

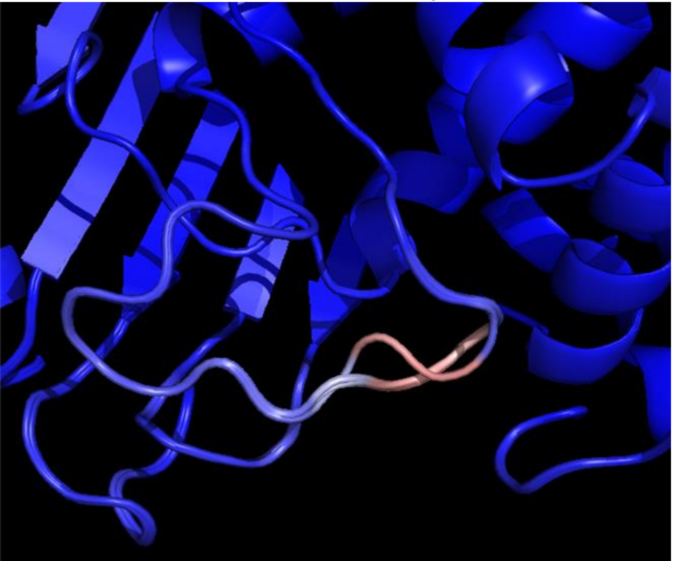

Color	pLDDT (Confidence)
Blue	High (90–100)
White	Medium (70–90)
Red	Low (<70)

PAE Heatmap for model 0

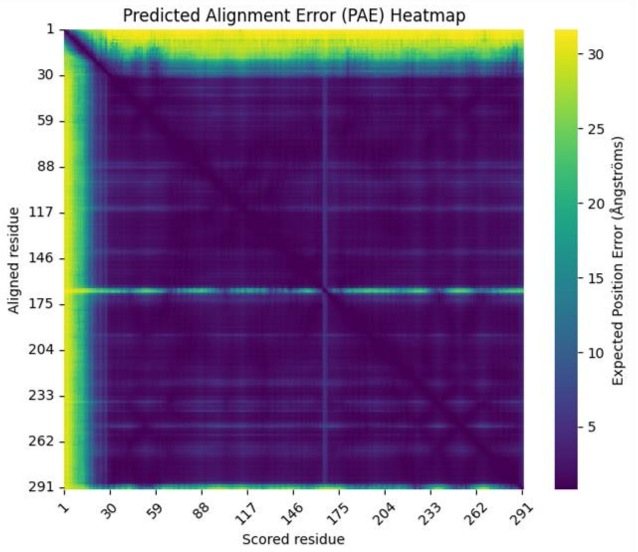

KPC-160

Model 0 was selected

AlphaFold model	pTM	pLDDT_global	pLDDT_region_ 164-170	RMSD vs model 0 (global)	RMSD vs model 0 (region 164-170)
Model 0	0.89	89.14	76.08	-	-
Model 1	0.89	88.91	75.00	0.067	0.660
Model 2	0.89	88.83	74.73	0.097	0.672
Model 3	0.89	88.80	74.79	0.070	0.650
Model 4	0.89	88.97	75.31	0.105	0.677

Models colored based on pLDDT scores

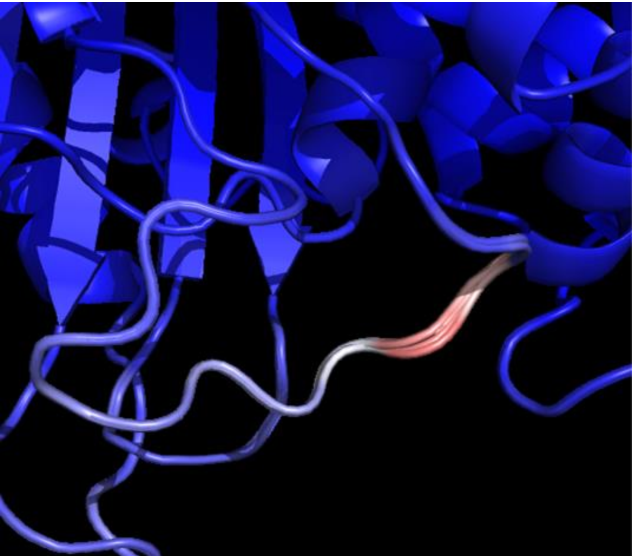

Color	pLDDT (Confidence)
Blue	High (90–100)
White	Medium (70–90)
Red	Low (<70)

PAE Heatmap for model 0

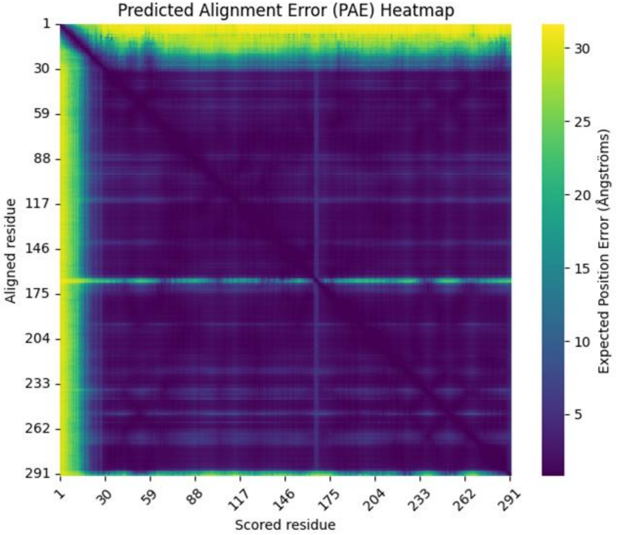

KPC-201

Model 1 was selected

AlphaFold model	pTM	pLDDT_global	pLDDT_region_ 159-169	RMSD vs model 1 (global)	RMSD vs model 1 (region 159-169)
Model 0	0.89	87.36	58.85	0.077	0.377
Model 1	0.88	87.43	60.08	-	-
Model 2	0.88	87.27	58.96	0.101	0.732
Model 3	0.89	87.45	59.85	0.075	0.360
Model 4	0.88	87.35	59.45	0.069	0.472

Models colored based on pLDDT scores

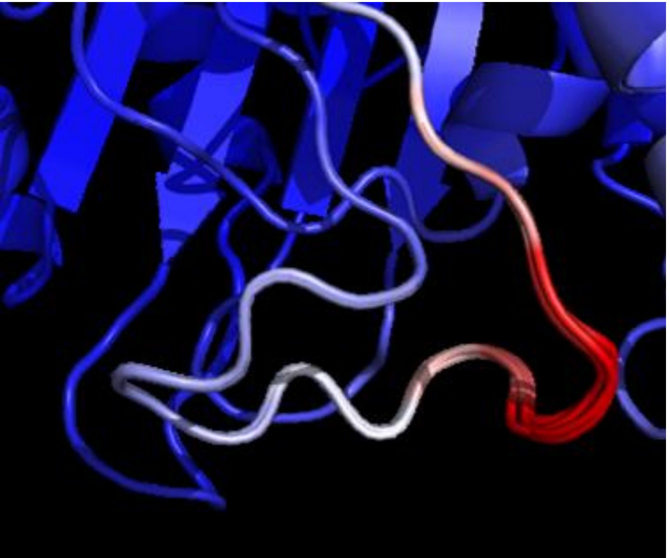

Color	pLDDT (Confidence)
Blue	High (90–100)
White	Medium (70–90)
Red	Low (<70)

PAE Heatmap for model 1

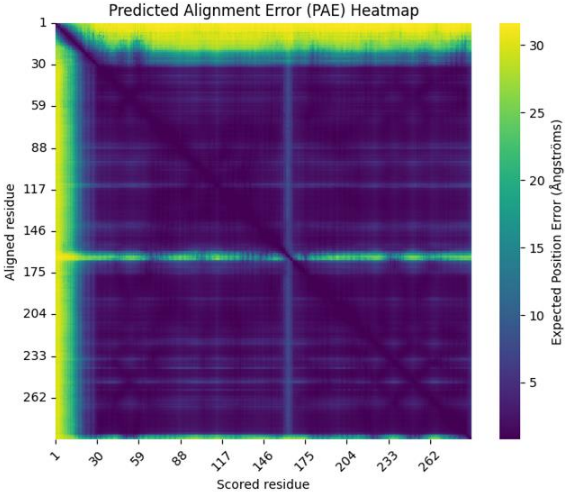

KPC-239

Model 1 was selected

AlphaFold model	pTM	pLDDT_global	pLDDT_region_ 237-243	RMSD vs model 1 (global)	RMSD vs model 1 (region 237-243)
Model 0	0.90	90.23	87.71	0.072	1.811
Model 1	0.90	90.15	88.98	-	-
Model 2	0.90	90.11	87.95	0.079	1.112
Model 3	0.90	90.03	87.57	0.072	1.568
Model 4	0.90	90.08	88.98	0.081	0.089

Models colored based on pLDDT scores

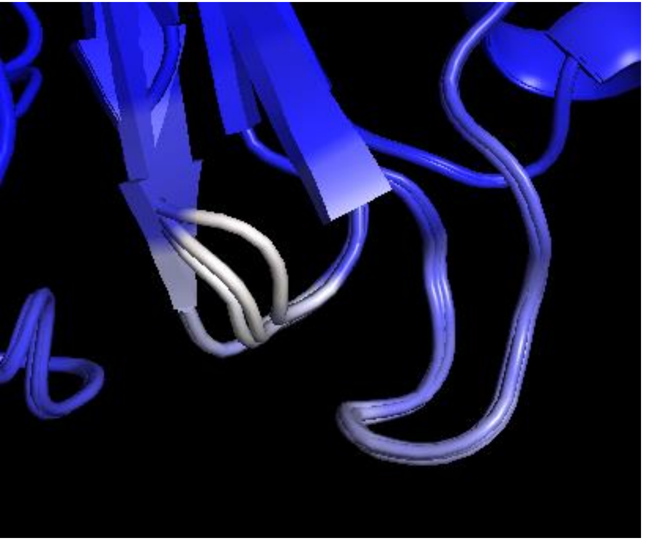

Color	pLDDT (Confidence)
Blue	High (90–100)
White	Medium (70–90)
Red	Low (<70)

PAE Heatmap for model 1

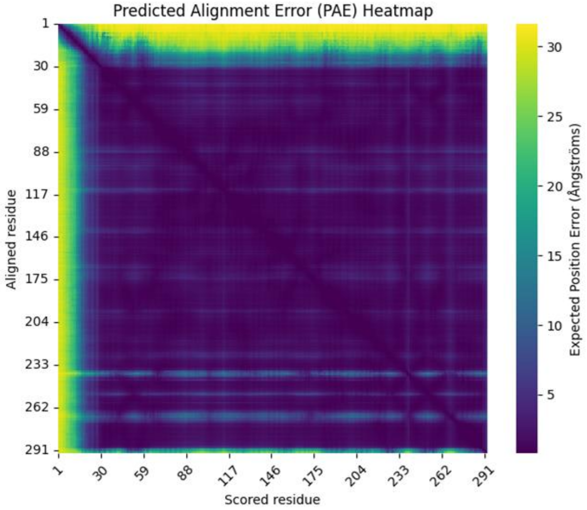

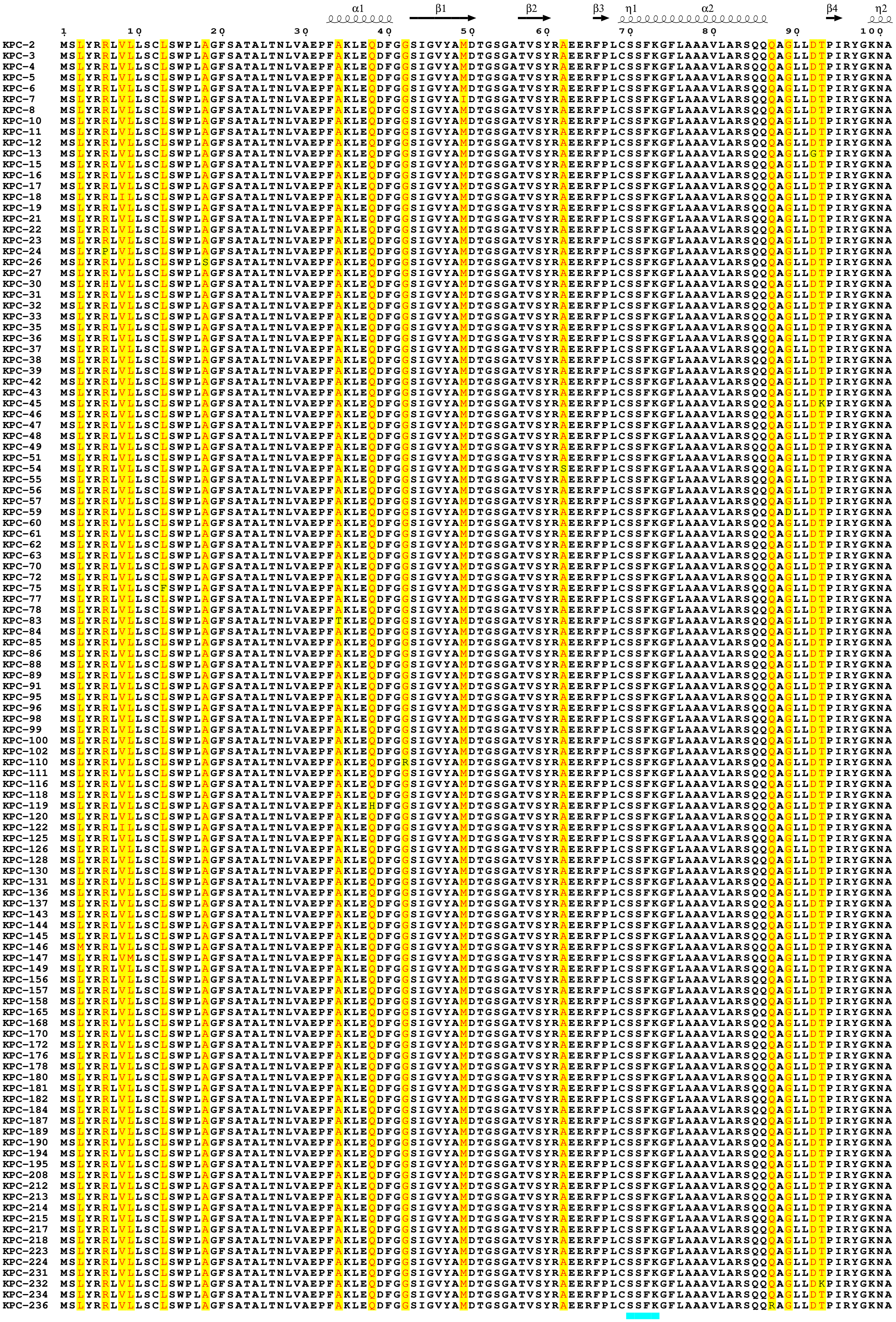
α1 β1 β2 β3 η1 α2 β4 η2
1 10 20 30 40 50 60 70 80 90 100
KPC-2 MSLYRRLVLLSCLSWPLAGFSATALTNLVAEPFAKLEQDFGGSIGVYAMDTGSGATVSYRAEERFPLCSSFKGFLAAAVLARSQQQAGLLDTPIRYGKNA
KPC-3 MSLYRRLVLLSCLSWPLAGFSATALTNLVAEPFAKLEQDFGGSIGVYAMDTGSGATVSYRAEERFPLCSSFKGFLAAAVLARSQQQAGLLDTPIRYGKNA
KPC-4 MSLYRRLVLLSCLSWPLAGFSATALTNLVAEPFAKLEQDFGGSIGVYAMDTGSGATVSYRAEERFPLCSSFKGFLAAAVLARSQQQAGLLDTPIRYGKNA
KPC-5 MSLYRRLVLLSCLSWPLAGFSATALTNLVAEPFAKLEQDFGGSIGVYAMDTGSGATVSYRAEERFPLCSSFKGFLAAAVLARSQQQAGLLDTPIRYGKNA
KPC-6 MSLYRRLVLLSCLSWPLAGFSATALTNLVAEPFAKLEQDFGGSIGVYAMDTGSGATVSYRAEERFPLCSSFKGFLAAAVLARSQQQAGLLDTPIRYGKNA
KPC-7 MSLYRRLVLLSCLSWPLAGFSATALTNLVAEPFAKLEQDFGGSIGVYAIDTGSGATVSYRAEERFPLCSSFKGFLAAAVLARSQQQAGLLDTPIRYGKNA
KPC-8 MSLYRRLVLLSCLSWPLAGFSATALTNLVAEPFAKLEQDFGGSIGVYAMDTGSGATVSYRAEERFPLCSSFKGFLAAAVLARSQQQAGLLDTPIRYGKNA
KPC-10 MSLYRRLVLLSCLSWPLAGFSATALTNLVAEPFAKLEQDFGGSIGVYAMDTGSGATVSYRAEERFPLCSSFKGFLAAAVLARSQQQAGLLDTPIRYGKNA
KPC-11 MSLYRRLVLLSCLSWPLAGFSATALTNLVAEPFAKLEQDFGGSIGVYAMDTGSGATVSYRAEERFPLCSSFKGFLAAAVLARSQQQAGLLDTPIRYGKNA
KPC-12 MSLYRRLVLLSCLSWPLAGFSATALTNLVAEPFAKLEQDFGGSIGVYAMDTGSGATVSYRAEERFPLCSSFKGFLAAAVLARSQQQAGLLDTPIRYGKNA
KPC-13 MSLYRRLVLLSCLSWPLAGFSATALTNLVAEPFAKLEQDFGGSIGVYAMDTGSGATVSYRAEERFPLCSSFKGFLAAAVLARSQQQAGLLGTPIRYGKNA
KPC-15 MSLYRRLVLLSCLSWPLAGFSATALTNLVAEPFAKLEQDFGGSIGVYAMDTGSGATVSYRAEERFPLCSSFKGFLAAAVLARSQQQAGLLDTPIRYGKNA
KPC-16 MSLYRRLVLLSCLSWPLAGFSATALTNLVAEPFAKLEQDFGGSIGVYAMDTGSGATVSYRAEERFPLCSSFKGFLAAAVLARSQQQAGLLDTPIRYGKNA
KPC-17 MSLYRRLVLLSCLSWPLAGFSATALTNLVAEPFAKLEQDFGGSIGVYAMDTGSGATVSYRAEERFPLCSSFKGFLAAAVLARSQQQAGLLDTPIRYGKNA
KPC-18 MSLYRRLILLSCLSWPLAGFSATALTNLVAEPFAKLEQDFGGSIGVYAMDTGSGATVSYRAEERFPLCSSFKGFLAAAVLARSQQQAGLLDTPIRYGKNA
KPC-19 MSLYRRLVLLSCLSWPLAGFSATALTNLVAEPFAKLEQDFGGSIGVYAMDTGSGATVSYRAEERFPLCSSFKGFLAAAVLARSQQQAGLLDTPIRYGKNA
KPC-21 MSLYRRLVLLSCLSWPLAGFSATALTNLVAEPFAKLEQDFGGSIGVYAMDTGSGATVSYRAEERFPLCSSFKGFLAAAVLARSQQQAGLLDTPIRYGKNA
KPC-22 MSLYRRLVLLSCLSWPLAGFSATALTNLVAEPFAKLEQDFGGSIGVYAMDTGSGATVSYRAEERFPLCSSFKGFLAAAVLARSQQQAGLLDTPIRYGKNA
KPC-23 MSLYRRLVLLSCLSWPLAGFSATALTNLVAEPFAKLEQDFGGSIGVYAMDTGSGATVSYRAEERFPLCSSFKGFLAAAVLARSQQQAGLLDTPIRYGKNA
KPC-24 MSLYRPLVLLSCLSWPLAGFSATALTNLVAEPFAKLEQDFGGSIGVYAMDTGSGATVSYRAEERFPLCSSFKGFLAAAVLARSQQQAGLLDTPIRYGKNA
KPC-26 MSLYRRLVLLSCLSWPLSGFSATALTNLVAEPFAKLEQDFGGSIGVYAMDTGSGATVSYRAEERFPLCSSFKGFLAAAVLARSQQQAGLLDTPIRYGKNA
KPC-27 MSLYRRLVLLSCLSWPLAGFSATALTNLVAEPFAKLEQDFGGSIGVYAMDTGSGATVSYRAEERFPLCSSFKGFLAAAVLARSQQQAGLLDTPIRYGKNA
KPC-30 MSLYRHLVLLSCLSWPLAGFSATALTNLVAEPFAKLEQDFGGSIGVYAMDTGSGATVSYRAEERFPLCSSFKGFLAAAVLARSQQQAGLLDTPIRYGKNA
KPC-31 MSLYRRLVLLSCLSWPLAGFSATALTNLVAEPFAKLEQDFGGSIGVYAMDTGSGATVSYRAEERFPLCSSFKGFLAAAVLARSQQQAGLLDTPIRYGKNA
KPC-32 MSLYRRLVLLSCLSWPLAGFSATALTNLVAEPFAKLEQDFGGSIGVYAMDTGSGATVSYRAEERFPLCSSFKGFLAAAVLARSQQQAGLLDTPIRYGKNA
KPC-33 MSLYRRLVLLSCLSWPLAGFSATALTNLVAEPFAKLEQDFGGSIGVYAMDTGSGATVSYRAEERFPLCSSFKGFLAAAVLARSQQQAGLLDTPIRYGKNA
KPC-35 MSLYRRLVLLSCLSWPLAGFSATALTNLVAEPFAKLEQDFGGSIGVYAMDTGSGATVSYRAEERFPLCSSFKGFLAAAVLARSQQQAGLLDTPIRYGKNA
KPC-36 MSLYRRLVLLSCLSWPLAGFSATALTNLVAEPFAKLEQDFGGSIGVYAMDTGSGATVSYRAEERFPLCSSFKGFLAAAVLARSQQQAGLLDTPIRYGKNA
KPC-37 MSLYRRLVLLSCLSWPLAGFSATALTNLVAEPFAKLEQDFGGSIGVYAMDTGSGATVSYRAEERFPLCSSFKGFLAAAVLARSQQQAGLLDTPIRYGKNA
KPC-38 MSLYRRLVLLSCLSWPLAGFSATALTNLVAEPFAKLEQDFGGSIGVYAMDTGSGATVSYRAEERFPLCSSFKGFLAAAVLARSQQQAGLLDTPIRYGKNA
KPC-39 MSLYRRLVLLSCLSWPLAGFSATALTNLVAEPFAKLEQDFGGSIGVYAMDTGSGATVSYRAEERFPLCSSFKGFLAAAVLARSQQQAGLLDTPIRYGKNA
KPC-42 MSLYRRLVLLSCLSWPLAGFSATALTNLVAEPFAKLEQDFGGSIGVYAMDTGSGATVSYRAEERFPLCSSFKGFLAAAVLARSQQQAGLLDTPIRYGKNA
KPC-43 MSLYRRLVLLSCLSWPLAGFSATALTNLVAEPFAKLEQDFGGSIGVYAMDTGSGATVSYRAEERFPLCSSFKGFLAAAVLARSQQQAGLLDTPIRYGKNA
KPC-45 MSLYRRLVLLSCLSWPLAGFSATALTNLVAEPFAKLEQDFGGSIGVYAMDTGSGATVSYRAEERFPLCSSFKGFLAAAVLARSQQQAGLLDKPIRYGKNA
KPC-46 MSLYRRLVLLSCLSWPLAGFSATALTNLVAEPFAKLEQDFGGSIGVYAMDTGSGATVSYRAEERFPLCSSFKGFLAAAVLARSQQQAGLLDTPIRYGKNA
KPC-47 MSLYRRLVLLSCLSWPLAGFSATALTNLVAEPFAKLEQDFGGSIGVYAMDTGSGATVSYRAEERFPLCSSFKGFLAAAVLARSQQQAGLLDTPIRYGKNA
KPC-48 MSLYRRLVLLSCLSWPLAGFSATALTNLVAEPFAKLEQDFGGSIGVYAMDTGSGATVSYRAEERFPLCSSFKGFLAAAVLARSQQQAGLLDTPIRYGKNA
KPC-49 MSLYRRLVLLSCLSWPLAGFSATALTNLVAEPFAKLEQDFGGSIGVYAMDTGSGATVSYRAEERFPLCSSFKGFLAAAVLARSQQQAGLLDTPIRYGKNA
KPC-51 MSLYRRLVLLSCLSWPLAGFSATALTNLVAEPFAKLEQDFGGSIGVYAMDTGSGATVSYRAEERFPLCSSFKGFLAAAVLARSQQQAGLLDTPIRYGKNA
KPC-54 MSLYRRLVLLSCLSWPLAGFSATALTNLVAEPFAKLEQDFGGSIGVYAMDTGSGATVSYRSEERFPLCSSFKGFLAAAVLARSQQQAGLLDTPIRYGKNA
KPC-55 MSLYRRLVLLSCLSWPLAGFSATALTNLVAEPFAKLEQDFGGSIGVYAMDTGSGATVSYRAEERFPLCSSFKGFLAAAVLARSQQQAGLLDTPIRYGKNA
KPC-56 MSLYRRLVLLSCLSWPLAGFSATALTNLVAEPFAKLEQDFGGSIGVYAMDTGSGATVSYRAEERFPLCSSFKGFLAAAVLARSQQQAGLLDTPIRYGKNA
KPC-57 MSLYRRLVLLSCLSWPLAGFSATALTNLVAEPFAKLEQDFGGSIGVYAMDTGSGATVSYRAEERFPLCSSFKGFLAAAVLARSQQQAGLLDTPIRYGKNA
KPC-59 MSLYRRLVLLSCLSWPLAGFSATALTNLVAEPFAKLEQDFGGSIGVYAMDTGSGATVSYRAEERFPLCSSFKGFLAAAVLARSQQQADLLDTPIRYGKNA
KPC-60 MSLYRRLVLLSCLSWPLAGFSATALTNLVAEPFAKLEQDFGGSIGVYAMDTGSGATVSYRAEERFPLCSSFKGFLAAAVLARSQQQAGLLDTPIRYGKNA
KPC-61 MSLYRRLVLLSCLSWPLAGFSATALTNLVAEPFAKLEQDFGGSIGVYAMDTGSGATVSYRAEERFPLCSSFKGFLAAAVLARSQQQAGLLDTPIRYGKNA
KPC-62 MSLYRRLVLLSCLSWPLAGFSATALTNLVAEPFAKLEQDFGGSIGVYAMDTGSGATVSYRAEERFPLCSSFKGFLAAAVLARSQQQAGLLDTPIRYGKNA
KPC-63 MSLYRRLVLLSCLSWPLAGFSATALTNLVAEPFAKLEQDFGGSIGVYAMDTGSGATVSYRAEERFPLCSSFKGFLAAAVLARSQQQAGLLDTPIRYGKNA
KPC-70 MSLYRRLVLLSCLSWPLAGFSATALTNLVAEPFAKLEQDFGGSIGVYAMDTGSGATVSYRAEERFPLCSSFKGFLAAAVLARSQQQAGLLDTPIRYGKNA
KPC-72 MSLYRRLVLLSCLSWPLAGFSATALTNLVAEPFAKLEQDFGGSIGVYAMDTGSGATVSYRAEERFPLCSSFKGFLAAAVLARSQQQAGLLDTPIRYGKNA
KPC-75 MSLYRRLVLLSCFSWPLAGFSATALTNLVAEPFAKLEQDFGGSIGVYAMDTGSGATVSYRAEERFPLCSSFKGFLAAAVLARSQQQAGLLDTPIRYGKNA
KPC-77 MSLYRRLVLLSCLSWPLAGFSATALTNLVAEPFAKLEQDFGGSIGVYAMDTGSGATVSYRAEERFPLCSSFKGFLAAAVLARSQQQAGLLDTPIRYGKNA
KPC-78 MSLYRRLVLLSCLSWPLAGFSATALTNLVAEPFAKLEQDFGGSIGVYAMDTGSGATVSYRAEERFPLCSSFKGFLAAAVLARSQQQAGLLDTPIRYGKNA
KPC-83 MSLYRRLVLLSCLSWPLAGFSATALTNLVAEPFTKLEQDFGGSIGVYAMDTGSGATVSYRAEERFPLCSSFKGFLAAAVLARSQQQAGLLDTPIRYGKNA
KPC-84 MSLYRRLVLLSCLSWPLAGFSATALTNLVAEPFAKLEQDFGGSIGVYAMDTGSGATVSYRAEERFPLCSSFKGFLAAAVLARSQQQAGLLDTPIRYGKNA
KPC-85 MSLYRRLVLLSCLSWPLAGFSATALTNLVAEPFAKLEQDFGGSIGVYAMDTGSGATVSYRAEERFPLCSSFKGFLAAAVLARSQQQAGLLDTPIRYGKNA
KPC-86 MSLYRRLVLLSCLSWPLAGFSATALTNLVAEPFAKLEQDFGGSIGVYAMDTGSGATVSYRAEERFPLCSSFKGFLAAAVLARSQQQAGLLDTPIRYGKNA
KPC-88 MSLYRRLVLLSCLSWPLAGFSATALTNLVAEPFAKLEQDFGGSIGVYAMDTGSGATVSYRAEERFPLCSSFKGFLAAAVLARSQQQAGLLDTPIRYGKNA
KPC-89 MSLYRRLVLLSCLSWPLAGFSATALTNLVAEPFAKLEQDFGGSIGVYAMDTGSGATVSYRAEERFPLCSSFKGFLAAAVLARSQQQAGLLDTPIRYGKNA
KPC-91 MSLYRRLVLLSCLSWPLAGFSATALTNLVAEPFAKLEQDFGGSIGVYAMDTGSGATVSYRAEERFPLCSSFKGFLAAAVLARSQQQAGLLDTPIRYGKNA
KPC-95 MSLYRRLVLLSCLSWPLAGFSATALTNLVAEPFAKLEQDFGGSIGVYAMDTGSGATVSYRAEERFPLCSSFKGFLAAAVLARSQQQAGLLDTPIRYGKNA
KPC-96 MSLYRRLVLLSCLSWPLAGFSATALTNLVAEPFAKLEQDFGGSIGVYAMDTGSGATVSYRAEERFPLCSSFKGFLAAAVLARSQQQAGLLDTPIRYGKNA
KPC-98 MSLYRRLVLLSCLSWPLAGFSATALTNLVAEPFAKLEQDFGGSIGVYAMDTGSGATVSYRAEERFPLCSSFKGFLAAAVLARSQQQAGLLDTPIRYGKNA
KPC-99 MSLYRRLVLLSCLSWPLAGFSATALTNLVAEPFAKLEQDFGGSIGVYAMDTGSGATVSYRAEERFPLCSSFKGFLAAAVLARSQQQAGLLDTPIRYGKNA
KPC-100 MSLYRRLVLLSCLSWPLAGFSATALTNLVAEPFAKLEQDFGGSIGVYAMDTGSGATVSYRAEERFPLCSSFKGFLAAAVLARSQQQAGLLDTPIRYGKNA
KPC-102 MSLYRRLVLLSCLSWPLAGFSATALTNLVAEPFAKLEQDFGGSIGVYAMDTGSGATVSYRAEERFPLCSSFKGFLAAAVLARSQQQAGLLDTPIRYGKNA
KPC-110 MSLYRRLVLLSCLSWPLAGFSATALTNLVAEPFAKLEQDFGRSIGVYAMDTGSGATVSYRAEERFPLCSSFKGFLAAAVLARSQQQAGLLDTPIRYGKNA
KPC-111 MSLYRRLVLLSCLSWPLAGFSATALTNLVAEPFAKLEQDFGGSIGVYAMDTGSGATVSYRAEERFPLCSSFKGFLAAAVLARSQQQAGLLDTPIRYGKNA
KPC-116 MSLYRRLVLLSCLSWPLAGFSATALTNLVAEPFAKLEQDFGGSIGVYAMDTGSGATVSYRAEERFPLCSSFKGFLAAAVLARSQQQAGLLDTPIRYGKNA
KPC-118 MSLYRRLVLLSCLSWPLAGFSATALTNLVAEPFAKLEQDFGGSIGVYAMDTGSGATVSYRAEERFPLCSSFKGFLAAAVLARSQQQAGLLDTPIRYGKNA
KPC-119 MSLYRRLVLLSCLSWPLAGFSATALTNLVAEPFAKLEHDFGGSIGVYAMDTGSGATVSYRAEERFPLCSSFKGFLAAAVLARSQQQAGLLDTPIRYGKNA
KPC-120 MSLYRRLVLLSCLSWPLAGFSATALTNLVAEPFAKLEQDFGGSIGVYAMDTGSGATVSYRAEERFPLCSSFKGFLAAAVLARSQQQAGLLDTPIRYGKNA
KPC-122 MSLYRRLILLSCLSWPLAGFSATALTNLVAEPFAKLEQDFGGSIGVYAMDTGSGATVSYRAEERFPLCSSFKGFLAAAVLARSQQQAGLLDTPIRYGKNA
KPC-125 MSLYRRLVLLSCLSWPLAGFSATALTNLVAEPFAKLEQDFGGSIGVYAMDTGSGATVSYRAEERFPLCSSFKGFLAAAVLARSQQQAGLLDTPIRYGKNA
KPC-126 MSLYRRLVLLSCLSWPLAGFSATALTNLVAEPFAKLEQDFGGSIGVYAMDTGSGATVSYRAEERFPLCSSFKGFLAAAVLARSQQQAGLLDTPIRYGKNA
KPC-128 MSLYRRLVLLSCLSWPLAGFSATALTNLVAEPFAKLEQDFGGSIGVYAMDTGSGATVSYRAEERFPLCSSFKGFLAAAVLARSQQQAGLLDTPIRYGKNA
KPC-130 MSLYRRLVLLSCLSWPLAGFSATALTNLVAEPFAKLEQDFGGSIGVYAMDTGSGATVSYRAEERFPLCSSFKGFLAAAVLARSQQQAGLLDTPIRYGKNA
KPC-131 MSLYRRLVLLSCLSWPLAGFSATALTNLVAEPFAKLEQDFGGSIGVYAMDTGSGATVSYRAEERFPLCSSFKGFLAAAVLARSQQQAGLLDTPIRYGKNA
KPC-136 MSLYRRLVLLSCLSWPLAGFSATALTNLVAEPFAKLEQDFGGSIGVYAMDTGSGATVSYRAEERFPLCSSFKGFLAAAVLARSQQQAGLLDTPIRYGKNA
KPC-137 MSLYRRLVLLSCLSWPLAGFSATALTNLVAEPFAKLEQDFGGSIGVYAMDTGSGATVSYRAEERFPLCSSFKGFLAAAVLARSQQQAGLLDTPIRYGKNA
KPC-143 MSLYRRLVLLSCLSWPLAGFSATALTNLVAEPFAKLEQDFGGSIGVYAMDTGSGATVSYRAEERFPLCSSFKGFLAAAVLARSQQQAGLLDTPIRYGKNA
KPC-144 MSLYRRLVLLSCLSWPLAGFSATALTNLVAEPFAKLEQDFGGSIGVYAMDTGSGATVSYRAEERFPLCSSFKGFLAAAVLARSQQQAGLLDTPIRYGKNA
KPC-145 MSLYRRLVLLSCLSWPLAGFSATALTNLVAEPFAKLEQDFGGSIGVYAMDTGSGATVSYRAEERFPLCSSFKGFLAAAVLARSQQQAGLLDTPIRYGKNA
KPC-146 MSMYRRLVLLSCLSWPLAGFSATALTNLVAEPFAKLEQDFGGSIGVYAMDTGSGATVSYRAEERFPLCSSFKGFLAAAVLARSQQQAGLLDTPIRYGKNA
KPC-147 MSLYRRLVMLSCLSWPLAGFSATALTNLVAEPFAKLEQDFGGSIGVYAMDTGSGATVSYRAEERFPLCSSFKGFLAAAVLARSQQQAGLLDTPIRYGKNA
KPC-149 MSLYRRLVLLSCLSWPLAGFSATALTNLVAEPFAKLEQDFGGSIGVYAMDTGSGATVSYRAEERFPLCSSFKGFLAAAVLARSQQQAGLLDTPIRYGKNA
KPC-156 MSLYRRLVLLSCLSWPLAGFSATALTNLVAEPFAKLEQDFGGSIGVYAMDTGSGATVSYRAEERFPLCSSFKGFLAAAVLARSQQQAGLLDTPIRYGKNA
KPC-157 MSLYRRLVLLSCLSWPLAGFSATALTNLVAEPFAKLEQDFGGSIGVYAMDTGSGATVSYRAEERFPLCSSFKGFLAAAVLARSQQQAGLLDTPIRYGKNA
KPC-158 MSLYRRLVLLSCLSWPLAGFSATALTNLVAEPFAKLEQDFGGSIGVYAMDTGSGATVSYRAEERFPLCSSFKGFLAAAVLARSQQQAGLLDTPIRYGKNA
KPC-165 MSLYRRLVLLSCLSWPLAGFSATALTNLVAEPFAKLEQDFGGSIGVYAMDTGSGATVSYRAEERFPLCSSFKGFLAAAVLARSQQQAGLLDTPIRYGKNA
KPC-168 MSLYRRLVLLSCLSWPLAGFSATALTNLVAEPFAKLEQDFGGSIGVYAMDTGSGATVSYRAEERFPLCSSFKGFLAAAVLARSQQQAGLLDTPIRYGKNA
KPC-170 MSLYRRLVLLSCLSWPLAGFSATALTNLVAEPFAKLEQDFGGSIGVYAMDTGSGATVSYRAEERFPLCSSFKGFLAAAVLARSQQQAGLLDTPIRYGKNA
KPC-172 MSLYRRLVLLSCLSWPLAGFSATALTNLVAEPFAKLEQDFGGSIGVYAMDTGSGATVSYRAEERFPLCSSFKGFLAAAVLARSQQQAGLLDTPIRYGKNA
KPC-176 MSLYRRLVLLSCLSWPLAGFSATALTNLVAEPFAKLEQDFGGSIGVYAMDTGSGATVSYRAEERFPLCSSFKGFLAAAVLARSQQQAGLLDTPIRYGKNA
KPC-178 MSLYRRLVLLSCLSWPLAGFSATALTNLVAEPFAKLEQDFGGSIGVYAMDTGSGATVSYRAEERFPLCSSFKGFLAAAVLARSQQQAGLLDTPIRYGKNA
KPC-180 MSLYRRLVLLSCLSWPLAGFSATALTNLVAEPFAKLEQDFGGSIGVYAMDTGSGATVSYRAEERFPLCSSFKGFLAAAVLARSQQQAGLLDTPIRYGKNA
KPC-181 MSLYRRLVLLSCLSWPLAGFSATALTNLVAEPFAKLEQDFGGSIGVYAMDTGSGATVSYRAEERFPLCSSFKGFLAAAVLARSQQQAGLLDTPIRYGKNA
KPC-182 MSLYRRLVLLSCLSWPLAGFSATALTNLVAEPFAKLEQDFGGSIGVYAMDTGSGATVSYRAEERFPLCSSFKGFLAAAVLARSQQQAGLLDTPIRYGKNA
KPC-184 MSLYRRLVLLSCLSWPLAGFSATALTNLVAEPFAKLEQDFGGSIGVYAMDTGSGATVSYRAEERFPLCSSFKGFLAAAVLARSQQQAGLLDTPIRYGKNA
KPC-187 MSLYRRLVLLSCLSWPLAGFSATALTNLVAEPFAKLEQDFGGSIGVYAMDTGSGATVSYRAEERFPLCSSFKGFLAAAVLARSQQQAGLLDTPIRYGKNA
KPC-189 MSLYRRLVLLSCLSWPLAGFSATALTNLVAEPFAKLEQDFGGSIGVYAMDTGSGATVSYRAEERFPLCSSFKGFLAAAVLARSQQQAGLLDTPIRYGKNA
KPC-190 MSLYRRLVLLSCLSWPLAGFSATALTNLVAEPFAKLEQDFGGSIGVYAMDTGSGATVSYRAEERFPLCSSFKGFLAAAVLARSQQQAGLLDTPIRYGKNA
KPC-194 MSLYRRLVLLSCLSWPLAGFSATALTNLVAEPFAKLEQDFGGSIGVYAMDTGSGATVSYRAEERFPLCSSFKGFLAAAVLARSQQQAGLLDTPIRYGKNA
KPC-195 MSLYRRLVLLSCLSWPLAGFSATALTNLVAEPFAKLEQDFGGSIGVYAMDTGSGATVSYRAEERFPLCSSFKGFLAAAVLARSQQQAGLLDTPIRYGKNA
KPC-208 MSLYRRLVLLSCLSWPLAGFSATALTNLVAEPFAKLEQDFGGSIGVYAMDTGSGATVSYRAEERFPLCSSFKGFLAAAVLARSQQQAGLLDTPIRYGKNA
KPC-212 MSLYRRLVLLSCLSWPLAGFSATALTNLVAEPFAKLEQDFGGSIGVYAMDTGSGATVSYRAEERFPLCSSFKGFLAAAVLARSQQQAGLLDTPIRYGKNA
KPC-213 MSLYRRLVLLSCLSWPLAGFSATALTNLVAEPFAKLEQDFGGSIGVYAMDTGSGATVSYRAEERFPLCSSFKGFLAAAVLARSQQQAGLLDTPIRYGKNA
KPC-214 MSLYRRLVLLSCLSWPLAGFSATALTNLVAEPFAKLEQDFGGSIGVYAMDTGSGATVSYRAEERFPLCSSFKGFLAAAVLARSQQQAGLLDTPIRYGKNA
KPC-215 MSLYRRLVLLSCLSWPLAGFSATALTNLVAEPFAKLEQDFGGSIGVYAMDTGSGATVSYRAEERFPLCSSFKGFLAAAVLARSQQQAGLLDTPIRYGKNA
KPC-217 MSLYRRLVLLSCLSWPLAGFSATALTNLVAEPFAKLEQDFGGSIGVYAMDTGSGATVSYRAEERFPLCSSFKGFLAAAVLARSQQQAGLLDTPIRYGKNA
KPC-218 MSLYRRLVLLSCLSWPLAGFSATALTNLVAEPFAKLEQDFGGSIGVYAMDTGSGATVSYRAEERFPLCSSFKGFLAAAVLARSQQQAGLLDTPIRYGKNA
KPC-223 MSLYRRLVLLSCLSWPLAGFSATALTNLVAEPFAKLEQDFGGSIGVYAMDTGSGATVSYRAEERFPLCSSFKGFLAAAVLARSQQQAGLLDTPIRYGKNA
KPC-224 MSLYRRLVLLSCLSWPLAGFSATALTNLVAEPFAKLEQDFGGSIGVYAMDTGSGATVSYRAEERFPLCSSFKGFLAAAVLARSQQQAGLLDTPIRYGKNA
KPC-231 MSLYRRLVLLSCLSWPLAGFSATALTNLVAEPFAKLEQDFGGSIGVYAMDTGSGATVSYRAEERFPLCSSFKGFLAAAVLARSQQQAGLLDTPIRYGKNA
KPC-232 MSLYRRLVLLSCLSWPLAGFSATALTNLVAEPFAKLEQDFGGSIGVYAMDTGSGATVSYRAEERFPLCSSFKGFLAAAVLARSQQQAGLLDKPIRYGKNA
KPC-234 MSLYRRLVLLSCLSWPLAGFSATALTNLVAEPFAKLEQDFGGSIGVYAMDTGSGATVSYRAEERFPLCSSFKGFLAAAVLARSQQQAGLLDTPIRYGKNA
KPC-236 MSLYRRLVLLSCLSWPLAGFSATALTNLVAEPFAKLEQDFGGSIGVYAMDTGSGATVSYRAEERFPLCSSFKGFLAAAVLARSQQRAGLLDTPIRYGKNA

```
              η3      β5   α3              α4               α5                          η4               β6    α6
              110       120       130       140       150       160       170       180       190       200
KPC-2    LVPWSPISEKYLTTGMTVAELSAAAVQYSDNAAANLLLKELGGPAGLTAFMRSIGDTTFRLDRWELELNSAIPGDARDTSSPRAVTESLQKLTLGSALAA
KPC-3    LVPWSPISEKYLTTGMTVAELSAAAVQYSDNAAANLLLKELGGPAGLTAFMRSIGDTTFRLDRWELELNSAIPGDARDTSSPRAVTESLQKLTLGSALAA
KPC-4    LVRWSPISEKYLTTGMTVAELSAAAVQYSDNAAANLLLKELGGPAGLTAFMRSIGDTTFRLDRWELELNSAIPGDARDTSSPRAVTESLQKLTLGSALAA
KPC-5    LVRWSPISEKYLTTGMTVAELSAAAVQYSDNAAANLLLKELGGPAGLTAFMRSIGDTTFRLDRWELELNSAIPGDARDTSSPRAVTESLQKLTLGSALAA
KPC-6    LVPWSPISEKYLTTGMTVAELSAAAVQYSDNAAANLLLKELGGPAGLTAFMRSIGDTTFRLDRWELELNSAIPGDARDTSSPRAVTESLQKLTLGSALAA
KPC-7    LVPWSPISEKYLTTGMTVAELSAAAVQYSDNAAANLLLKELGGPAGLTAFMRSIGDTTFRLDRWELELNSAIPGDARDTSSPRAVTESLQKLTLGSALAA
KPC-8    LVPWSPISEKYLTTGMTVAELSAAAVQYSDNAAANLLLKELGGPAGLTAFMRSIGDTTFRLDRWELELNSAIPGDARDTSSPRAVTESLQKLTLGSALAA
KPC-10   LVRWSPISEKYLTTGMTVAELSAAAVQYSDNAAANLLLKELGGPAGLTAFMRSIGDTTFRLDRWELELNSAIPGDARDTSSPRAVTESLQKLTLGSALAA
KPC-11   LVLWSPISEKYLTTGMTVAELSAAAVQYSDNAAANLLLKELGGPAGLTAFMRSIGDTTFRLDRWELELNSAIPGDARDTSSPRAVTESLQKLTLGSALAA
KPC-12   LVPWSPISEKYLTTGMTVAELSAAAVQYSDNAAANLLLKELGGPAGLTAFMRSIGDTTFRLDRWELEMNSAIPGDARDTSSPRAVTESLQKLTLGSALAA
KPC-13   LVPWSPISEKYLTTGMTVAELSAAAVQYSDNAAANLLLKELGGPAGLTAFMRSIGDTTFRLDRWELELNSAIPGDARDTSSPRAVTESLQKLTLGSALAA
KPC-15   LVRWSPISEKYLTTGMTVLELSAAAVQYSDNAAANLLLKELGGPAKLTAFMRSIGDTTFRLDRWELELNSAIPGDARDTSSPRAVTESLQKLTLGSALAA
KPC-16   LVPWSPISEKYLTTGMTVAELSAAAVQYSDNAAANLLLKELGGPAGLTAFMRSIGDTTFRLDRWELELNSAIPGDARDTSSPRAVTESLQKLTLGSALAA
KPC-17   LVPWSPISEKYLTTGMTVAELSAAAVQYSDNAAANLLLKELGGPAGLTAFMRSIGDTTFRLDRWELELNSAIPGDARDTSSPRAVTESLQKLTLGSALAA
KPC-18   LVPWSPISEKYLTTGMTVAELSAAAVQYSDNAAANLLLKELGGPAGLTAFMRSIGDTTFRLDRWELELNSAIPGDARDTSSPRAVTESLQKLTLGSALAA
KPC-19   LVPWSPISEKYLTTGMTVAELSAAAVQYSDNAAANLLLKELGGPAGLTAFMRSIGDTTFRLDRWELELNSAIPGDARDTSSPRAVTESLQKLTLGSALAA
KPC-21   LVPRSPISEKYLTTGMTVAELSAAAVQYSDNAAANLLLKELGGPAGLTAFMRSIGDTTFRLDRWELELNSAIPGDARDTSSPRAVTESLQKLTLGSALAA
KPC-22   LVPGSPISEKYLTTGMTVAELSAAAVQYSDNAAANLLLKELGGPAGLTAFMRSIGDTTFRLDRWELELNSAIPGDARDTSSPRAVTESLQKLTLGSALAA
KPC-23   LVPWSPISEKYLTTGMTVAELSAAAVQYSDNAAANLLLKELGGPAGLTAFMRSIGDTTFRLDRWELELNSAIPGDARDTSSPRAVTESLQKLTLGSALAA
KPC-24   LVPWSPISEKYLTTGMTVAELSAAAVQYSDNAAANLLLKELGGPAGLTAFMRSIGDTTFRLDRWELELNSAIPGDARDTSSPRAVTESLQKLTLGSALAA
KPC-26   LVPWSPISEKYLTTGMTVAELSAAAVQYSDNAAANLLLKELGGPAGLTAFMRSIGDTTFRLDRWELELNSAIPGDARDTSSPRAVTESLQKLTLGSALAA
KPC-27   LVPRSPISEKYLTTGMTVAELSAAAVQYSDNAAANLLLKELGGPAGLTAFMRSIGDTTFRLDRWELELNSAIPGDARDTSSPRAVTESLQKLTLGSALAA
KPC-30   LVPWSPISEKYLTTGMTVAELSAAAVQYSDNAAANLLLKELGGPAGLTAFMRSIGDTTFRLDRWELELNSAIPGDARDTSSPRAVTESLQKLTLGSALAA
KPC-31   LVPWSPISEKYLTTGMTVAELSAAAVQYSDNAAANLLLKELGGPAGLTAFMRSIGDTTFRLDRWELELNSAIPGDARYTSSPRAVTESLQKLTLGSALAA
KPC-32   LVPWSPISEKYLTTGMTVAELSAAAVQYSDNAAANLLLKELGGPAGLTAFMRSIGDTTFRLDRWELELNSAIPGDARYTSSPRAVTESLQKLTLGSALAA
KPC-33   LVPWSPISEKYLTTGMTVAELSAAAVQYSDNAAANLLLKELGGPAGLTAFMRSIGDTTFRLDRWELELNSAIPGDARYTSSPRAVTESLQKLTLGSALAA
KPC-35   LVPWSPISEKYLTTGMTVAELSAAAVQYSDNAAANLLLKELGGPAGLTAFMRSIGDTTFRLDRWELEPNSAIPGDARDTSSPRAVTESLQKLTLGSALAA
KPC-36   LVPWSPISEKYLTTGMTVAELSAAAVQYSDNAAANLLLKELGGPAGLTAFMRSIGDTTFRLERWELELNSAIPGDARDTSSPRAVTESLQKLTLGSALAA
KPC-37   LVPWSPISEKYLTTGMTVAELSAAAVQYSDNAAANLLLKELGGPAGLTAFMRSIGDTTFRLDRRELELNSAIPGDARDTSSPRAVTESLQKLTLGSALAA
KPC-38   LVPWSPISEKYLTTGMTVAELSAAAVQYSDNAAANLLLKELGGPAGLTAFMRSIGDTTFRLDRWELELNSAIPGDARDTSSPRAVTESLQKLTLGSALAA
KPC-39   LVPWSPISEKYLTTGMTVAELSAAAVQYSDNAAANLLLKELGGPAGLTAFMRSIGDTTFRLDRWELELNSTIPGDARDTSSPRAVTESLQKLTLGSALAA
KPC-42   LVPWSPISEKYLTTGMTVAELSAAAVQYSDNAAANLLLKELGGPAGLTAFMRSIGDTTFRLDRWELELNSAIPGDARDTSSPRAVTESLQKLTLGSALAA
KPC-43   LVPWSPISEKYLTTGMTVAELSAAAVQYSDNAAANLLLKELGGPAGLTAFMRSIGDTTFRLDRWELELNSAIPGDARDTSSPRAVTESLRKLTLGSALAA
KPC-45   LVPWSPISEKYLTTGMTVAELSAAAVQYSDNAAANLLLKELGGPAGLTAFMRSIGDTTFRLDRWELELNSAIPGDARDTSSPRAVTESLQKLTLGSALAA
KPC-46   LVPWSPISEKYLTTGMTVAELSAAAVQYSDNAAANLLLKELGGPAGLTAFMRSIGDTTFRLDRWELEPNSAIPGDARDTSSPRAVTESLQKLTLGSALAA
KPC-47   LVPWSPISEKYLTTGMTVAELSAAAVQYSDNAAANLLLKELGGPAGLTAFMRSIGDTTFRLDRWELELNSTIPGDARDTSSPRAVTESLQKLTLGSALAA
KPC-48   LVPWSPISEKYLTTGMTVAELSAAAVQYSDNAAANLLLKELGGPAGLTAFMRSIGDTTFRLDRWELEPNSTIPGDARDTSSPRAVTESLQKLTLGSALAA
KPC-49   LVPWSPISEKYLTTGMTVAELSAAAVQYSDNAAANLLLKELGGPAGLTAFMRSIGDTTFRLDSWELELNSAIPGDARDTSSPRAVTESLQKLTLGSALAA
KPC-51   LVPWSPISEKYLTTGMTVAELSAAAVQYSDNAAANLLLKELGGPAGLTAFMRSIGDTTFRLDRWELELNSAIPGDARNTSSPRAVTESLQKLTLGSALAA
KPC-54   LVPWSPISEKYLTTGMTVAELSAAAVQYSDNAAANLLLKELGGPAGLTAFMRSIGDTTFRLDRWELELNSAIPGDARDTSSPRAVTESLQKLTLGSALAA
KPC-55   LVPWSPISEKYLTTGMTVAELSAAAVQYSDNAAANLLLKELGGPAGLTAFMRSIGDTTFRLDRWELELNSAIPGDARDTSSPRAVTESLQKLTLGSALAA
KPC-56   LVPWSPISEKYLTTGMTVAELSAAAVQYSDNAAANLLLKELGGPAGLTAFMRSIGDTTFRLDRWELELNSAIPGDARDTSSPRAVTESLQKLTLGSALAA
KPC-57   LVPWSPISEKYLTTGMTVAELSAAAVQYSDNAAANLLLKELGGPAGLTAFMRSIGDTTFRLDRWELELNSAIPGDARVTSSPRAVTESLQKLTLGSALAA
KPC-59   LVPWSPISEKYLTTGMTVAELSAAAVQYSDNAAANLLLKELGGPAGLTAFMRSIGDTTFRLDRWELELNSAIPGDARDTSSPRAVTESLQKLTLGSALAA
KPC-60   LVPWSPISEKYLTTGMTVTELSAAAVQYSDNAAANLLLKELGGPAGLTAFMRSIGDTTFRLDRWELELNSAIPGDARDTSSPRAVTESLQKLTLGSALAA
KPC-61   LVPWSPISEKYLTTGMTVAELSAAAVQYSDNAAANLLLKELGGPAGLTAFMRSIGDTTFRLDRWELELNPAIPGDARDTSSPRAVTESLQKLTLGSALAA
KPC-62   LVPWSPISEKYLTTGMTVAELSAAAVQYSDNAAANLLLKELGGPAGLTAFMRSIGDTTFRLDRWELEQNSAIPGDARDTSSPRAVTESLQKLTLGSALAA
KPC-63   LVPWSPISEKYLTTGMTVAELSAAAVQYSDNAAANLLLKELGGPAGLTAFMRSIGDTTFRLDRWELEQNSAIPGDARDTSSPRAVTESLQKLTLGSALAA
KPC-70   LVPWSPISEKYLTTGMTVAELSAAAVQYSDNAAANLLLKELGGPAGLTAFMRSIGDTTFRLDRWELELNSAIPGDARYTSSPRAVTESLQKLTLGSALAA
KPC-72   LVPWSPISEKYLTTGMTVAELSAAAVQYSDNAAANLLLKELGGPAGLTAFMRSIGDTTFRLDRWELELNSDIPGDARDTSSPRAVTESLQKLTLGSALAA
KPC-75   LVPWSPISEKYLTTGMTVAELSAAAVQYSDNAAANLLLKELGGPAGLTAFMRSIGDTTFRLDRWELELNSAIPGDARDTSSPRAVTESLQKLTLGSALAA
KPC-77   LVPWSPISEKYLTTGMTVAELSAAAVQYSDNAAANLLLKELGGPAGLTAFMRSIGDTTFRLDPWELELNSAIPGDARDTSSPRAVTESLQKLTLGSALAA
KPC-78   LVPWSPISEKYLTTGMTVAELSAAAVQYSDNAAANLLLKELGGPAGLTAFMRSIGDTTFRLDRWELELNSAIPGDARATSSPRAVTESLQKLTLGSALAA
KPC-83   LVPWSPISEKYLTTGMTVAELSAAAVQYSDNAAANLLLKELGGPAGLTAFMRSIGDTTFRLDRWELELNSAIPGDARDTSSPRAVTESLQKLTLGSALAA
KPC-84   LVPWSPISEKYLTTGMTVAELSAAAVQYSDNAAANLLLKELGGPAGLTAFMRSIGDTTFRLDRWELELNSAIPGDARDTSSPRAVTESLQKLTLGSALAA
KPC-85   LVPWSPISEKYLTTGMTVAELSAAAVQYSDNAAANLLLKELGGPAGLTAFMRSIGDTTFRLDRWELELNSVIPGDARDTSSPRAVTESLQKLTLGSALAA
KPC-86   LVPWSPISEKYLTTGMTVAELSAAAVQYSDNAAANLLLKELGGPAGLTAFMRSIGDTTFRLDRWELELNSAIPGDARGTSSPRAVTESLQKLTLGSALAA
KPC-88   LVPWSPISEKYLTTGMTVAELSAAAVQYSDNAAANLLLKELGGPAGLTAFMRSIGDTTFRLDRWELELNSAIPGYARDTSSPRAVTESLQKLTLGSALAA
KPC-89   LVPWSPISEKYLTTGMTVAELSAAAVQYSDNAAANLLLKELGGPAGLTAFMRSIGDTTFRLDRWELELNSAIPGDARDTSSPRAVTESLQKLTLGSALAA
KPC-91   LLPWSPISEKYLTTGMTVAELSAAAVQYSDNAAANLLLKELGGPAGLTAFMRSIGDTTFRLDRWELELNSAIPGDARDTSSPRAVTESLQKLTLGSALAA
KPC-95   LVPWSPISEKYLTTGMTVAELSAAAVQYSDNAAANLLLKELGGPAGLTAFMRSIGDTTFRLDRWELELNSTIPGDARYTSSPRAVTESLQKLTLGSALAA
KPC-96   LVPWSPISEKYLTTGMTVAELSAAAVQYSDNAAANLLLKELGGPAGLTAFMRSIGDTTFRLDRWELELNSAIPGDARDTSSPRAVTESLQKLTLGSALAA
KPC-98   LVPWSPISEKYLTTGMTVAELSAAAVQYSDNAAANLLLKELGGPAGLTAFMRSIGDTTFRLDHWELELNSAIPGDARDTSSPRAVTESLQKLTLGSALAA
KPC-99   LVPWSPISEKYLTTGMTVAELSAAAVQYSDNAAANLLLKELGGPAGLTAFMRSIGDTTFRLDSWELELNSAIPGDARDTSSPRAVTESLQKLTLGSALAA
KPC-100  LVPWSPISEKYLTTGMTVAELSAAAVQYSDNAAANLLLKELGGPAGLTAFMRSIGDTTFRLDRWELELNSAIPGDARDTSSPRAVTESLQKLTLGSALAA
KPC-102  LVPWSPISEKYLTTGMTVAELSAAAVQYSDNAAANLLLKELGGPAGLTAFMRSIGDTTFRLDRWELELNSAIPGDARYTSSPRAVTESLQKLTLGSALAA
KPC-110  LVPWSPISEKYLTTGMTVAELSAAAVQYSDNAAANLLLKELGGPAGLTAFMRSIGDTTFRLDRWELELNSAIPGDARYTSSPRAVTESLQKLTLGSALAA
KPC-111  LVPWSPISEKYLTTGMTVAELSAAAVQYSDNAAANLLLKELGGPAGLTAFMRSIGDTTFRLDRWELELNSAILGDARYTSSPRAVTESLQKLTLGSALAA
KPC-116  LVPWSPISEKYLTTGMTVAELSAAAVQYSDNAAANLLLKELGGPAGLTAFMRSIGDTTFRLDRWELELNFAIPGDARDTSSPRAVTESLQKLTLGSALAA
KPC-118  LLPWSPISEKYLTTGMTVAELSAAAVQYSDNAAANLLLKELGGPAGLTAFMRSIGDTTFRLDRRELELNSAIPGDARDTSSPRAVTESLQKLTLGSALAA
KPC-119  LVPWSPISEKYLTTGMTVAELSAAAVQYSDNAAANLLLKELGGPAGLTAFMRSIGDTTFRLDRWELELNSAIPGDARDTSSPRAVTESLQKLTLGSALAA
KPC-120  LVRWSPISEKYLTTGMTVAELSAAAVQYSDNAAANLLLKELGGPAGLTAFMRSIGDTTFRLDRWELELNSAIPGDARYTSSPRAVTESLQKLTLGSALAA
KPC-122  LVPWSPISEKYLTTGMTVAELSAAAVQYSDNAAANLLLKELGGPAGLTAFMRSIGDTTFRLDRWELELNSAIPGDARDTSSPRAVTESLQKLTLGSALAA
KPC-125  LVPWSPISEKYLTTGMTVAELSAAAVQYSDNAAANLLLKELGGPAGLTAFMRSIGDTTFRLDRWELELNSAIPGDARATSSPRAVTESLQKLTLGSALAA
KPC-126  LVPWSPISEKYLTTGMTVAELSAAAVQYSDNAAANLLLKELGGPAGLTAFMRSIGDTTFRLDRWELELNSVIPGDARYTSSPRAVTESLQKLTLGSALAA
KPC-128  LVPWSPISEKYLTTGMTVAELSAAAVQYSDNAAANLLLKELGGPAGLTAFMRSIGDTTFRLDRWELELNSAIPGDARYTSSPRAVTESLQKLTLGSALAA
KPC-130  LVPWSPISEKYLTTGMTVAELSAAAVQYSDNAAANLLLKELGGPAGLTAFMRSIGDTTFRLDRWELELNSAIPGDARGTSSPRAVTESLQKLTLGSALAA
KPC-131  LVPWSPISEKYLTTGMTVAELSAAAVQYSDNAAANLLLKELGGPAGLTAFMRSIGDTTFRLDRWELELNSAIPGDARDTSSPRAVTESLQKLTLGSALAA
KPC-136  LVPWSPISEKYLTTGMTVAELSAAAVQYSDNAAANLLLKELGGPAGLTAFMRSIGDTTFRLDRWELELNSAILGDARDTSSPRAVTESLQKLTLGSALAA
KPC-137  LVPWSPISEKYLTTGMTVAELSAAAVQYSDNAAANLLLKELGGPAGLTAFMRSIGDTTFRLDRWELELNSAILDDARDTSSPRAVTESLQKLTLGSALAA
KPC-143  LVPWSPISEKYLTTGMTVAELSAAAVQYSDNAAANLLLKELGGPAGLTAFMRSIGDTTFRLDRWELELNSAIPGDARYTSSPRAVSESLQKLTLGSALAA
KPC-144  LVPWSPISEKYLTTGMTVAELSAAAVQYSDNAAANLLLKELGGPAGLTAFMRSIGDTTFRLDRWELELNSTIPGDARDTSSPRAVTESLQKLTLGSALAA
KPC-145  LVPWSPISEKYLTTGMTVAELSAAAVQYSDNAAANLLLKELGGPAGLTAFMRSIGDTTFRLDRWELELNSAIPGDARYTSSPRAVTESLQKLTLGSALAA
KPC-146  LVPWSPISEKYLTTGMTVAELSAAAVQYSDNAAANLLLKELGGPAGLTAFMRSIGDTTFRLDRWELELNSAIPGDARDTSSPRAVTESLQKLTLGSALAA
KPC-147  LVPWSPISEKYLTTGMTVAELSAAAVQYSDNAAANLLLKELGGPAGLTAFMRSIGDTTFRLDRWELELNSAIPGDARDTSSPRAVTESLQKLTLGSALAA
KPC-149  LVPWSPISEKYLTTGMTVAELSAAAVQYSDNAAANLLLKELGGPAGLTAFMRSIGDTTFRLDRWELELNSAIPGDARDTSSPRAVTESLQKLTLGSALAA
KPC-156  LVPWSPISEKYLTTGMTVAELSAAAVQYSDNAAANLLLKELGGPAGLTAFMRSIGDTTFRLDRWELELNSAIPGDARDTSSPRAVTESLQKLTLGSALAA
KPC-157  LVPWSPISEKYLTTGMTVAELSAAAVQYSDSAAANLLLKELGGPAGLTAFMRSIGDTTFRLDRWELELNSAIPGDARDTSSPRAVTESLQKLTLGSALAA
KPC-158  LVPWSPISEKYLTTGMTVAELSAAAVQYSDNAAANLLLKELGGPAGLTAFMRSIGDTTFRLDRWELELNSAIPGDARDTSSPRAVTESLQKLTLGSALAA
KPC-165  LVPWSPISEKYLTTGMTVAELSAAAVQYSDNAAANLLLKELGGPAGLTAFMRSIGDTTFRLDRWELELDSAIPGDARDTSSPRAVTESLQKLTLGSALAA
KPC-168  LVPWSPISEKYLTTGMTVAELSAAAVQYSDNAAANLLLKELGGPAGLTAFMRSIGDTTFRLDRWELELNSAIPGNARDTSSPRAVTESLQKLTLGSALAA
KPC-170  LVPWSPISEKYLTTGMTVAELSAAAVQYSDNAAANLLLKELGGPAGLTAFMRSIGDTTFRLDRWELELNSAIPGDARNTSSPRAVTESLQKLTLGSALAA
KPC-172  LVPWSPISEKYLTTGMTVAELSAAAVQYSDNAAANLLLKELGGPAGLTAFMRSIGDTTFRLDRWELELNSAIPGDARETSSPRAVTESLQKLTLGSALAA
KPC-176  LVPWSPISEKYLTTGMTVAELSAAAVQYSDNAAANLLLKELGGPAGLTAFMRSIGDTTFRLDRWELELNPAIPGDARDTSSPRAVTESLQKLTLGSALAA
KPC-178  LVPWSPISEKYLTTGMTVAELSAAAVQYSDNAAANLLLKELGGPAGLTAFMRSIGDTTFRLDRWELELNSAILGDARDTSSPRAVTESLQKLTLGSALAA
KPC-180  LVPWSPISEKYLTTGMTVAELSAAAVQYSDNAAANLLLKELGGPAGLTAFMRSIGDTTFRLDHWELELNSAIPGDARDTSSPRAVTESLQKLTLGSALAA
KPC-181  LVPWSPISEKYLTTGMTVAELSAAAVQYSDNAAANLLLKELGGPAGLTAFMRSIGDTTFRLDRWELELNSAIPGDARDTSSPRAVTESLQKLTLGSALAA
KPC-182  LVPWSPISEKYLTTGMTVAELSAAAVQYSDNAAANLLLKELGGPAGLTAFMRSIGDTTFRLDRWELELNSAIPGDARDTSSPRAVTESLQKLTLGSALAA
KPC-184  LVPWSPISEKYLTTGMTVAELSAAAVQYSDNAAANLLLKELGGPAGLTAFMRSIGDTTFRLDRWELELNSAIPGDARDTSSPRAVTESLQKLTLGSALAA
KPC-187  LVPWSPISEKYLTTGMTVAELSAAAVQYSDNAAANLLLKELGGPAGLTAFMRSIGDTTFRLDHWELELNSAIPGDARYTSSPRAVTESLQKLTLGSALAA
KPC-189  LVPWSPISEKYLTTGMTVAELSAAAVQYSDNAAANLLLKELGGPAGLTAFMRSIGDTTFRLDRWELELNSTIPGDARYTSSPRAVTESLQKLTLGSALAA
KPC-190  LVPWSPISEKYLTTGMTVAELSAAAVQYSDNAAANLLLKELGGPAGLTAFMRSIGDTTFRLDRWELELNSAIPGDARYTSSPRAVTESLQKLTLGSALAA
KPC-194  LVPWSPISEKYLTTGMTVAELSAAAVQYSDNAAANLLLKELGGPAGLTAFMRSIGDTTFRLDRWELELNSAIPGDARYTSSLRAVTESLQKLTLGSALAA
KPC-195  LVPWSPISEKYLTTGMTVAELSAAAVQYSDNAAANLLLKELGGPAGLTAFMRSIGDTTFRLDRWELELNSAIPGDARDTSSPRAVTESLQKLTLGSALAA
KPC-208  LVPWSPISEKYLTTGMTVAELSAAAVQYSDNAAANLLLKELGGPAGLTAFMRSIGDTTFRLDRWELEQNSAIPGDARYTSSPRAVTESLQKLTLGSALAA
KPC-212  LVPWSPISEKYLTTGMTVAELSAAAVQYSDNAAANLLLKELGGPAGLTAFMRSIGDTTFRLDLWELELNSAIPGDARDTSSPRAVTESLQKLTLGSALAA
KPC-213  LVPWSPISEKYLTTGMTVAELSAAAVQYSDNAAANLLLKELGGPAGLTAFMRSIGDTTFRLGRWELELNSAIPGDARDTSSPRAVTESLQKLTLGSALAA
KPC-214  LVPWSPISEKYLTTGMTVAELSAAAVQYSDNAAANLLLKELGGPAGLTAFMRSIGDTTFRLDRWELELNSAIPGDAHDTSSPRAVTESLQKLTLGSALAA
KPC-215  LVPWSPISEKYLTTGMTVAELSAAAVQYSDNAAANLLLKELGGPAGLTAFMRSIGDTTFRLDRWELELNSASPGDARDTSSPRAVTESLQKLTLGSALAA
KPC-217  LVPWSPISEKYLTTGMTVAELSAAAVQYSDNAAANLLLKELGGPAGLTAFMRSIGDTTFRLDRWELELNSDIPGDARDTSSPRAVTESLQKLTLGSALAA
KPC-218  LVPWSPISEKYLTTGMTVAELSAAAVQYSDNAAANLLLKELGGPAGLTAFMRSIGDTTFRLDRWELELNSAILGDARDTSSPRAVTESLQKLTLGSALAA
KPC-223  LVPWSPISEKYLTTGMTVAELSAAAVQYSDNAAANLLLKELGGPAGLTAFMRSIGDTTFRLDRWELELNSAIPGDARDTSSPRAVTESLQKLTLGSALAA
KPC-224  LVPWSPISEKYLTTGMTVAELSAAAVQYSDNAAANLLLKELGGPAGLTAFMRSIGDTTFRLDRWELEQNSAIPGDARDTSSPRAVTESLQKLTLGSALAA
KPC-231  LVPWSPISEKYLTTGMTVAELSAAAVQYSDNAAANLLLKELGGPAGLTAFMRSIGDTTFRLDRWELELNYAIPGDARDTSSPRAVTESLQKLTLGSALAA
KPC-232  LVPWSPISEKYLTTGMTVAELSAAAVQYSDNAAANLLLKELGGPAGLTAFMRSIGDTTFRLDRWELELNSAIPGDARDTSSPRAVTESLQKLTLGSALAA
KPC-234  LVPWSPISEKYLTTGMTVAELSAAAVQYSDNAAANLLLKELGGPAGLTAFMRSIGDTTFRLDRWELELNSAIPGDARGTSSPRAVTESLQKLTLGSALAA
KPC-236  LVPWSPISEKYLTTGMTVAELSAAAVQYSDNAAANLLLKELGGPAGLTAFMRSIGDTTFRLDRWELELNSAIPGDARDTSSPRAVTESLQKLTLGSALAA
```

```
             α7            η5        β7          β8          β9              α8
               210        220        230        240        250                 270        280        290
KPC-2    PQRQQFVDWLKGNTTGNHRIRAAVPADWAVGDKTGTCGVYGTANDYAVVWPTGRAPIVLAVYTRAPNKDDKHSEAVIAAAARLALEGLGVNGQ
KPC-3    PQRQQFVDWLKGNTTGNHRIRAAVPADWAVGDKTGTCGVYGTANDYAVVWPTGRAPIVLAVYTRAPNKDDKYSEAVIAAAARLALEGLGVNGQ
KPC-4    PQRQQFVDWLKGNTTGNHRIRAAVPADWAVGDKTGTCGGYGTANDYAVVWPTGRAPIVLAVYTRAPNKDDKHSEAVIAAAARLALEGLGVNGQ
KPC-5    PQRQQFVDWLKGNTTGNHRIRAAVPADWAVGDKTGTCGVYGTANDYAVVWPTGRAPIVLAVYTRAPNKDDKHSEAVIAAAARLALEGLGVNGQ
KPC-6    PQRQQFVDWLKGNTTGNHRIRAAVPADWAVGDKTGTCGGYGTANDYAVVWPTGRAPIVLAVYTRAPNKDDKHSEAVIAAAARLALEGLGVNGQ
KPC-7    PQRQQFVDWLKGNTTGNHRIRAAVPADWAVGDKTGTCGVYGTANDYAVVWPTGRAPIVLAVYTRAPNKDDKYSEAVIAAAARLALEGLGVNGQ
KPC-8    PQRQQFVDWLKGNTTGNHRIRAAVPADWAVGDKTGTCGGYGTANDYAVVWPTGRAPIVLAVYTRAPNKDDKYSEAVIAAAARLALEGLGVNGQ
KPC-10   PQRQQFVDWLKGNTTGNHRIRAAVPADWAVGDKTGTCGVYGTANDYAVVWPTGRAPIVLAVYTRAPNKDDKYSEAVIAAAARLALEGLGVNGQ
KPC-11   PQRQQFVDWLKGNTTGNHRIRAAVPADWAVGDKTGTCGVYGTANDYAVVWPTGRAPIVLAVYTRAPNKDDKHSEAVIAAAARLALEGLGVNGQ
KPC-12   PQRQQFVDWLKGNTTGNHRIRAAVPADWAVGDKTGTCGVYGTANDYAVVWPTGRAPIVLAVYTRAPNKDDKHSEAVIAAAARLALEGLGVNGQ
KPC-13   PQRQQFVDWLKGNTTGNHRIRAAVPADWAVGDKTGTCGVYGTANDYAVVWPTGRAPIVLAVYTRAPNKDDKYSEAVIAAAARLALEGLGVNGQ
KPC-15   PQRQQFVDWLKGNTTGNHRIRAAVPADWAVGDKTGTCGGYGTANDYAVVWPTGRAPIVLAVYTRAPNKDDKYSEAVIAAAARLALEGLGVNGQ
KPC-16   SQRQQLVDWLKGNTTGNHRIRAAVPADWAVGDKTGTCGVYGTANDYAVVWPTGRAPIVLAVYTRAPNKDDKHSEAVIAAAARLALEGLGVNGQ
KPC-17   PQRQQLVDWLKGNTTGNHRIRAAVPADWAVGDKTGTCGVYGTANDYAVVWPTGRAPIVLAVYTRAPNKDDKHSEAVIAAAARLALEGLGVNGQ
KPC-18   PQRQQFVDWLKGNTTGNHRIRAAVPADWAVGDKTGTCGVYGTANDYAVVWPTGRAPIVLAVYTRAPNKDDKHSEAVIAAAARLALEGLGVNGQ
KPC-19   PQRQQFVDWLKGNTTGNHRIRAAVPADWAVGDKTGTCGVYGTANDYAVVWPTGRAPIVLAVYTRAPNKDDKYSEAVIAAAARLALEGLGVTGQ
KPC-21   PQRQQFVDWLKGNTTGNHRIRAAVPADWAVGDKTGTCGVYGTANDYAVVWPTGRAPIVLAVYTRAPNKDDKHSEAVIAAAARLALEGLGVNGQ
KPC-22   PQRQQLVDWLKGNTTGNHRIRAAVPADWAVGDKTGTCGVYGTANDYAVVWPTGRAPIVLAVYTRAPNKDDKHSEAVIAAAARLALEGLGVNGQ
KPC-23   PQRQQFVDWLKGNTTGNHRIRAAVPADWAVGDKTGTCGAYGTANDYAVVWPTGRAPIVLAVYTRAPNKDDKYSEAVIAAAARLALEGLGVNGQ
KPC-24   PQRQQFVDWLKGNTTGNHRIRAAVPADWAVGDKTGTCGVYGTANDYAVVWPTGRAPIVLAVYTRAPNKDDKHSEAVIAAAARLALEGLGVNGQ
KPC-26   PQRQQFVDWLKGNTTGNHRIRAAVPADWAVGDKTGTCGVYGTANDYAVVWPTGRAPIVLAVYTRAPNKDDKHSEAVIAAAARLALEGLGVNGQ
KPC-27   PQRQQFVDWLKGNTTGNHRIRAAVPADWAVGDKTGTCGVYGTANDYAVVWPTGRAPIVLAVYTRAPNKDDKYSEAVIAAAARLALEGLGVNGQ
KPC-30   PQRQQFVDWLKGNTTGNHRIRAAVPADWAVGDKTGTCGVYGTANDYAVVWPTGRAPIVLAVYTRAPNKDDKHSEAVIAAAARLALEGLGVNGQ
KPC-31   PQRQQFVDWLKGNTTGNHRIRAAVPADWAVGDKTGTCGVYGTANDYAVVWPTGRAPIVLAVYTRAPNKDDKYSEAVIAAAARLALEGLGVNGQ
KPC-32   PQRQQFVDWLKGNTTGNHRIRAAVPADWAVGDKTGTCGVYGMANDYAVVWPTGRAPIVLAVYTRAPNKDDKYSEAVIAAAARLALEGLGVNGQ
KPC-33   PQRQQFVDWLKGNTTGNHRIRAAVPADWAVGDKTGTCGVYGTANDYAVVWPTGRAPIVLAVYTRAPNKDDKHSEAVIAAAARLALEGLGVNGQ
KPC-35   PQRQQFVDWLKGNTTGNHRIRAAVPADWAVGDKTGTCGVYGTANDYAVVWPTGRAPIVLAVYTRAPNKDDKHSEAVIAAAARLALEGLGVNGQ
KPC-36   PQRQQFVDWLKGNTTGNHRIRAAVPADWAVGDKTGTCGVYGTANDYAVVWPTGRAPIVLAVYTRAPNKDDKYSEAVIAAAARLALEGLGVNGQ
KPC-37   PQRQQLVDWLKGNTTGNHRIRAAVPADWAVGDKTGTCGVYGTANDYAVVWPTGRAPIVLAVYTRAPNKDDKHSEAVIAAAARLALEGLGVNGQ
KPC-38   PQRQQFVDWLKGNTTGNHRIRAAVPADWAVGDKTGTCGVYGTANDYAVVWPTGRAPIVLAVYTRAPNKDDKYSEAVIAAAARLALEGLGANGQ
KPC-39   PQRQQFVDWLKGNTTGNHRIRAAVPADWAVGDKTGTCGVYGTANDYAVVWPTGRAPIVLAVYTRAPNKDDKYSEAVIAAAARLALEGLGVNGQ
KPC-42   PQRQQFVDWLKGNTTGNHRIRAAVPADWAVGDKTGTCGVYGTANDYAVVWPAGRAPIVLAVYTRAPNKDDKHSEAVIAAAARLALEGLGVNGQ
KPC-43   PQRQQFVDWLKGNTTGNHRIRAAVPADWAVGDKTGTCGVYGTANDYAVVWPTGRAPIVLAVYTRAPNKDDKHSEAVIAAAARLALEGLGVNGQ
KPC-45   PQRQQFVDWLKGNTTGNHRIRAAVPADWAVGDKTGTCGVYGTANDYAVVWPTGRAPIVLAVYTRAPNKDDKHSEAVIAAAARLALEGLGVNGQ
KPC-46   PQRQQFVDWLKGNTTGNHRIRAAVPADWAVGDKTGTCGVYGTANDYAVVWPTGRAPIVLAVYTRAPNKDDKYSEAVIAAAARLALEGLGVNGQ
KPC-47   PQRQQFVDWLKGNTTGNHRIRAAVPADWAVGDKTGTCGVYGAANDYAVVWPTGRAPIVLAVYTRAPNKDDKYSEAVIAAAARLALEGLGVNGQ
KPC-48   PQRQQFVDWLKGNTTGNHRIRAAVPADWAVGDKTGTCGVYGTANDYAVVWPTGRAPIVLAVYTRAPNKDDKYSEAVIAAAARLALEGLGVNGQ
KPC-49   PQRQQFVDWLKGNTTGNHRIRAAVPADWAVGDKTGTCGVYGTANDYAVVWPTGRAPIVLAVYTRAPNKDDKYSEAVIAAAARLALEGLGVNGQ
KPC-51   PQRQQFVDWLKGNTTGNHRIRAAVPADWAVGDKTGTCGVHGTANDYAVVWPTGRAPIVLAVYTRAPNKDDKNSEAVIAAAARLALEGLGVNGQ
KPC-54   PQRQQFVDWLKGNTTGNHRIRAAVPADWAVGDKTGTCGVYGTANDYAVVWPTGRAPIVLAVYTRAPNKDDKHSEAVIAAAARLALEGLGVNGQ
KPC-55   PQRQQFVDWLKGNTTGNHRIRAAVPADWAVGDKTGTCGVYGTANDYAVVWPTGRAPIVLAVNTRAPNKDDKHSEAVIAAAARLALEGLGVNGQ
KPC-56   PQRQQFVDWLKGNTTGNHRIRAAVPADWAVGDKTGTCGVYGTANDYAVVWPTGRAPIVLAVYTRAPNKDDKYSEAVIAAAARLALEGLGVNWQ
KPC-57   PQRQQFVDWLKGNTTGNHRIRAAVPADWAVGDKTGTCGVYGTANDYAVVWPTGRAPIVLAVYTRAPNKDDKHSEAVIAAAARLALEGLGVNGQ
KPC-59   PQRQQFVDWLKGNTTGNHRIRAAVPADWAVGDKTGTCGVYGTANDYAVVWPTGRAPIVLAVYTRAPNKDDKHSEAVIAAAARLALEGLGVNGQ
KPC-60   PQRQQFVDWLKGNTTGNHRIRAAVPADWAVGDKTGTCGVYGTANDYAVVWPTGRAPIVLAVYTRAPNKDDKHSEAVIAAAARLALEGLGVNGQ
KPC-61   PQRQQFVDWLKGNTTGNHRIRAAVPADWAVGDKTGTCGVYGTANDYAVVWPTGRAPIVLAVYTRAPNKDDKYSEAVIAAAARLALEGLGVNGQ
KPC-62   PQRQQFVDWLKGNTTGNHRIRAAVPADWAVGDKTGTCGVYGTANDYAVVWPTGRAPIVLAVYTRAPNKDDKYSEAVIAAAARLALEGLGVNGQ
KPC-63   PQRQQFVDWLKGNTTGNHRIRAAVPADWAVGDKTGTCGVSGTANDYAVVWPTGRAPIVLAVYTRAPNKDDKYSEAVIAAAARLALEGLGVNGQ
KPC-70   PQRQQFVDWLKGNTTGNHRIRAAVPADWAVGDKTGTCGVYGTANDYAVVWPTGRAPIVLAVYARAPNKDDKYSEAVIAAAARLALEGLGVNGQ
KPC-72   PQRQQFVDWLKGNTTGNHRIRAAVPADWAVGDKTGTCGVYGTANDYAVVWPTGRAPIVLAVYTRAPNKDDKHSEAVIAAAARLALEGLGVNGQ
KPC-75   PQRQQFVDWLKGNTTGNHRIRAAVPADWAVGDKTGTCGVYGTANDYAVVWPTGRAPIVLAVYTRAPNKDDKHSEAVIAAAARLALEGLGVNGQ
KPC-77   PQRQQFVDWLKGNTTGNHRIRAAVPADWAVGDKTGTCGVYGTANDYAVVWPTGRAPIVLAVYTRAPNKDDKHSEAVIAAAARLALEGLGVNGQ
KPC-78   PQRQQFVDWLKGNTTGNHRIRAAVPADWAVGDKTGTCGVYGTANDYAVVWPTGRAPIVLAVYTRAPNKDDKHSEAVIAAAARLALEGLGVNGQ
KPC-83   PQRQQFVDWLKGNTTGNHRIRAAVPADWAVGDKTGTCGVYGTANDYAVVWPTGRAPIVLAVYTRAPNKDDKHSEAVIAAAARLALEGLGVNGQ
KPC-84   PQRQQFVDWLKGNTTGNHRIRAAVPADWAVGDKTGTCGVYGPANDYAVVWPTGRAPIVLAVYTRAPNKDDKHSEAVIAAAARLALEGLGVNGQ
KPC-85   PQRQQFVDWLKGNTTGNHRIRAAVPADWAVGDKTGTCGVYGTANDYAVVWPTGRAPIVLAVYTRAPNKDDKYSEAVIAAAARLALEGLGVNGQ
KPC-86   PQRQQFVDWLKGNTTGNHRIRAAVPADWAVGDKTGTCGVYGTANDYAVVWPTGRAPIVLAVYTRAPNKDDKHSEAVIAAAARLALEGLGVNGQ
KPC-88   PQRQQFVDWLKGNTTGNHRIRAAVPADWAVGDKTGTCGVYGTANDYAVVWPTGRAPIVLAVYTRAPNKDDKHSEAVIAAAARLALEGLGVNGQ
KPC-89   PQRQQFVDWLKGNTTGNHRIRAAVPADWAVGDKTGTCGVYGMANDYAVVWPTGRAPIVLAVYTRAPNKDDKYSEAVIAAAARLALEGLGVNGQ
KPC-91   PQRQQFVDWLKGNTTGNHRIRAAVPADWAVGDKTGTCGVYGTANDYAVVWPTGRAPIVLAVYTRAPNKDDKYSEAVIAAAARLALEGLGVNGQ
KPC-95   PQRQQFVDWLKGNTTGNHRIRAAVPADWAVGDKTGTCGVYGTANDYAVVWPTGRAPIVLAVYTRAPNKDDKYSEAVIAAAARLALEGLGVNGQ
KPC-96   PQRQQFVDWLKGNTTGNHRIRAAVPADWAVGDKTGTCGVNGTANDYAVVWPTGRAPIVLAVYTRAPNKDDKHSEAVIAAAARLALEGLGVNGQ
KPC-98   PQRQQFVDWLKGNTTGNHRIRAAVPADWAVGDKTGTCGVYGTANDYAVVWPTGRAPIVLAVYTRAPNKDDKYSEAVIAAAARLALEGLGVNGQ
KPC-99   PQRQQFVDWLKGNTTGNHRIRAAVPADWAVGDKTGTCGVYGTANDYAVVWPTGRAPIVLAVYTRAPNKDDKHSEAVIAAAARLALEGLGVNGQ
KPC-100  PQRQQFVDWLKGNTTGNHRIRAAVPADWAVGDKTGTCGVYGMANDYAVVWPTGRAPIVLAVYTRAPNKDDKHSEAVIAAAARLALEGLGVNGQ
KPC-102  PQRQQFVDWLKGNTTGNHRIRAAVPADWAVGDKTGTCGVDGTANDYAVVWPTGRAPIVLAVYTRAPNKDDKHSEAVIAAAARLALEGLGVNGQ
KPC-110  PQRQQFVDWLKGNTTGNHRIRAAVPADWAVGDKTGTCGVYGTANDYAVVWPTGRAPIVLAVYTRAPNKDDKYSEAVIAAAARLALEGLGVNGQ
KPC-111  PQRQQFVDWLKGNTTGNHRIRAAVPADWAVGDKTGTCGVYGTANDYAVVWPTGRAPIVLAVYTRAPNKDDKYSEAVIAAAARLALEGLGVNGQ
KPC-116  PQRQQFVDWLKGNTTGNHRIRAAVPADWAVGDKTGTCGVYGTANDYAVVWPTGRAPIVLAVYTRAPNKDDKHSEAVIAAAARLALEGLGVNGQ
KPC-118  PQRQQFVDWLKGNTTGNHRIRAAVPADWAVGDKTGTCGVYGTANDYAVVWPTGRAPIVLAVYTRAPNKDDKYSEAVIAAAARLALEGLGVNGQ
KPC-119  PQRQQFVDWLKGNTTGNHRIRAAVPADWAVGDKTGTCGVYGTANDYAVVWPTGRAPIVLAVYTRAPNKDDKHSEAVIAAAARLALEGLGVNGQ
KPC-120  PQRQQFVDWLKGNTTGNHRIRAAVPADWAVGDKTGTCGVYGTANDYAVVWPTGRAPIVLAVYTRAPNKDDKHSEAVIAAAARLALEGLGVNGQ
KPC-122  PQRQQFVDWLKGNTTGNHRIRAAVPADWAVGDKTGTCGVYGTANDYAVVWPTGRAPIVLAVYTRAPNKDDKYSEAVIAAAARLALEGLGVNGQ
KPC-125  PQRQQFVDWLKGNTTGNHRIRAAVPADWAVGDKTGTCGVYGTANDYAVVWPTGRAPIVLAVYTRAPNKDDKYSEAVIAAAARLALEGLGVNGQ
KPC-126  PQRQQFVDWLKGNTTGNHRIRAAVPADWAVGDKTGTCGVYGTANDYAVVWPTGRAPIVLAVYTRAPNKDDKHSEAVIAAAARLALEGLGVNGQ
KPC-128  PQRQQFVDWLKGNTTGNHRIRAAVPADWAVGDKTGTCGVYGMANDYAVVWPTGRAPIVLAVYTRAPNKDDKHSEAVIAAAARLALEGLGVNGQ
KPC-130  PQRQQFVDWLKGNTTGNHRIRAAVPADWAVGDKTGTCGVYGTANDYAVVWPTGRAPIVLAVYTRAPNKDDKYSEAVIAAAARLALEGLGVNGQ
KPC-131  PQRQQFVDWLKGNTAGNHRIRAAVPADWAVGDKTGTCGVYGTANDYAVVWPTGRAPIVLAVYTRAPNKDDKHSEAVIAAAARLALEGLGVNGQ
KPC-136  PQRQQFVDWLKGNTTGNHRIRAAVPADWAVGDKTGTCGVYGTANDYAVVWPTGRAPIVLAVYTRAPNKDDKHSEAVIAAAARLALEGLGVNGQ
KPC-137  PQRQQFVDWLKGNTTGNHRIRAAVPADWAVGDKTGTCGVYGTANDYAVVWPTGRAPIVLAVYTRAPNKDDKHSEAVIAAAARLALEGLGVNGQ
KPC-143  PQRQQFVDWLKGNTTGNHRIRAAVPADWAVGDKTGTCGVYGTANDYAVVWPTGRAPIVLAVYTRAPNKDDKHSEAVIAAAARLALEGLGVNGQ
KPC-144  PQRQQFVDWLKGNTTGNHRIRAAVPADWAVGDKTGTCGVYGTANDYAVVWPTGRAPIVLAVYTRAPNKDDKHSEAVIAAAARLALEGLGVNGQ
KPC-145  PQRQQFVDWLKGNTTGNHRIRAAVPADWAVGDKTGTCGVYGTANDYAVVWPTGRAPIVLAVYARAPNKDDKHSEAVIAAAARLALEGLGVNGQ
KPC-146  PQRQQFVDWLKGNTTGNHRIRAAVPADWAVGDKTGTCGVYGTANDYAVVWPTGRAPIVLAVYTRAPNKDDKHSEAVIAAAARLALEGLGVNGQ
KPC-147  PQRQQFVDWLKGNTTGNHRIRAAVPADWAVGDKTGTCGVYGTANDYAVVWPTGRAPIVLAVYTRAPNKDDKHSEAVIAAAARLALEGLGVNGQ
KPC-149  PQRQQFVDWLKGSTTGNHRIRAAVPADWAVGDKTGTCGVYGTANDYAVVWPTGRAPIVLAVYTRAPNKDDKYSEAVIAAAARLALEGLGVNGQ
KPC-156  PQRQQFVDWLKGNTTGNHRIRAAVPADWAVGDKTGTCGVYGTANDYAVVWPTGRAPIVLAVYTRAPNKDDKHSEAVIAAAARLALEGLDVNGQ
KPC-157  PQRQQFVDWLKGNTTGNHRIRAAVPADWAVGDKTGTCGVYGTANDYAVVWPTGRAPIVLAVYTRAPNKDDKHSEAVIAAAARLALEGLGVNGQ
KPC-158  PQRQQFVDWLKGNTTGNHRIRAAVPADWAVGDKTGTCGVYGTVNDYAVVWPTGRAPIVLAVYTRAPNKDDKHSEAVIAAAARLALEGLGVNGQ
KPC-165  PQRQQFVDWLKGNTTGNHRIRAAVPADWAVGDKTGTCGVYGTANDYAVVWPTGRAPIVLAVYTRAPNKDDKHSEAVIAAAARLALEGLGVNGQ
KPC-168  PQRQQFVDWLKGNTTGNHRIRAAVPADWAVGDKTGTCGVYGTANDYAVVWPTGRAPIVLAVYTRAPNKDDKHSEAVIAAAARLALEGLGVNGQ
KPC-170  PQRQQFVDWLKGNTTGNHRIRAAVPADWAVGDKTGTCGVYGTANDYAVVWPTGRAPIVLAVYTRAPNKDDKHSEAVIAAAARLALEGLGVNGQ
KPC-172  PQRQQFVDWLKGNTTGNHRIRAAVPADWAVGDKTGTCGVYGTANDYAVVWPTGRAPIVLAVYTRAPNKDDKHSEAVIAAAARLALEGLGVNGQ
KPC-176  PQRQQFVDWLKGNTTGNHRIRAAVPADWAVGDKTGTCGVYGTANDYAVVWPTGRAPIVLAVYTRAPNKDDKHSEAVIAAAARLALEGLGVNGQ
KPC-178  PQRQQFVDWLKGNTTGNHRIRAAVPADWAVGDKTGTCGVYGTANDYAVVWPTGRAPIVLAVYTRAPNKDDKYSEAVIAAAARLALEGLGVNGQ
KPC-180  PQRQQFVDWLKGNTTGNHRIRAAVPADWAVGDKTGTCGVYGTANDYAVVWPTGRAPIVLAVYTRAPNKDDKHSEAVIAAAARLALEGLGVNGQ
KPC-181  PQRQQFVDWLKGNTTGNHRIRAAVPADWAVGDKTGTCGVYGTANDYAVVWPTGRAPIVLAVYTRAPNKDDKHSDAVIAAAARLALEGLGVNGQ
KPC-182  PQRQQFVDWLKGNTTGNHRIRAAVPADWAVGDKTGTCGVYGTANDYAVVWPTGRAPIVLAVYTRAPNKDDKHSEAVIAAAARLAIEGLGVNGQ
KPC-184  PQRQQFVDWLKGNTTGNHRIRAAVPADWAVGDKTGTCGVYGTANDYAVVWPTGRAPIVLAVYTRAPNKDDKYSDAVIAAAARLALEGLGVNGQ
KPC-187  PQRQQFVDWLKGNTTGNHRIRAAVPADWAVGDKTGTCGVYGTANDYAVVWPTGRAPIVLAVYTRAPNKDDKHSEAVIAAAARLALEGLGVNGQ
KPC-189  PQRQQFVDWLKGNTTGNHRIRAAVPADWAVGDKTGTCGVYGTANDYAVVWPTGRAPIVLAVYTRAPNKDDKHSEAVIAAAARLALEGLGVNGQ
KPC-190  PQRQQFVDWLKGNTTGNHRIRAAVPADWAVGDKTGTCGVYGTVNDYAVVWPTGRAPIVLAVYTRAPNKDDKHSEAVIAAAARLALEGLGVNGQ
KPC-194  PQRQQFVDWLKGNTTGNHRIRAAVPADWAVGDKTGTCGVYGTANDYAVVWPTGRAPIVLAVYTRAPNKDDKHSEAVIAAAARLALEGLGVNGQ
KPC-195  PQRQQFVDWLKGNTTGNHRIRAAVPADWAVGDKTGTCGVYGTANDYAVVWPTGRAPIVLAVYTRAPNKDDKHSEAVIAAAARLALDGLGVNGQ
KPC-208  PQRQQFVDWLKGNTTGNHRIRAAVPADWAVGDKTGTCGVYGTANDYAVVWPTGRAPIVLAVYTRAPNKDDKHSEAVIAAAARLALEGLGVNGQ
KPC-212  PQRQQFVDWLKGNTTGNHRIRAAVPADWAVGDKTGTCGVYGTANDYAVVWPTGRAPIVLAVYTRAPNKDDKHSEAVIAAAARLALEGLGVNGQ
KPC-213  PQRQQFVDWLKGNTTGNHRIRAAVPADWAVGDKTGTCGVYGTANDYAVVWPTGRAPIVLAVYTRAPNKDDKHSEAVIAAAARLALEGLGVNGQ
KPC-214  PQRQQFVDWLKGNTTGNHRIRAAVPADWAVGDKTGTCGVYGTANDYAVVWPTGRAPIVLAVYTRAPNKDDKHSEAVIAAAARLALEGLGVNGQ
KPC-215  PQRQQFVDWLKGNTTGNHRIRAAVPADWAVGDKTGTCGVYGTANDYAVVWPTGRAPIVLAVYTRAPNKDDKHSEAVIAAAARLALEGLGVNGQ
KPC-217  PQRQQFVDWLKGNTTGNHRIRAAVPADWAVGDKTGTCGVYGTANDYAVVWPTGRAPIVLAVYTRAPNKDDKYSDAVIAAAARLALEGLGVTGQ
KPC-218  PQRQQFVDWLKGNTTGNHRIRAAVPADWAVGDKTGTCGVYGTANDYAVVWPTGRAPIVLAVYTRAPNKDDKYSDAVIAAAARLALEGLGVTGQ
KPC-223  PQRQQFVDWLKGNTTGNHRIRAAVPADWAVGDKTGTCGVYGTANDYAVFWPTGRAPIVLAVYTRAPNKDDKHSEAVIAAAARLALEGLGVNGQ
KPC-224  PQRQQFVDWLKGNTTGNHRIRAAVPADWAVGDKTGTCGVYGTANDYAVVWPTGRAPIVLAVYTRAPNKDDKHSEAVIAAAARLALEGLGVNGQ
KPC-231  PQRQQFVDWLKGNTTGNHRIRAAVPADWAVGDKTGTCGVYGTANDYAVVWPTGRAPIVLAVYTRAPNKDDKYSEAVIAAAARLALEGLGVNGQ
KPC-232  PQRQQFVDWLKGNTTGNHRIRAAVPADWAVGDKTGTCGVYGTANDYAVVWPTGRAPIVLAVYTRAPNKDDKYSEAVIAAAARLALEGLGVNGQ
KPC-234  PQRQQFVDWLKGNTTGNHRIRAAVPADWAVGDKTGTCGVDGTANDYAVVWPTGRAPIVLAVYTRAPNKDDKHSEAVIAAAARLALEGLGVNGQ
KPC-236  PQRQQFVDWLKGNTTGNHRIRAAVPADWAVGDKTGTCGVYGTANDYAVVWPTGRAPIVLAVYTRAPNKDDKHSEAVIAAAARLALEGLGVNGQ
```
